# Supplementary figures and images for: Broad-scale factors shaping the ecological niche and geographic distribution of Spirodela polyrhiza
Source: PLoS One. 2023 May 4;18(5):e0276951. doi: 10.1371/journal.pone.0276951 (PMC10159170; doi:10.1371/journal.pone.0276951)

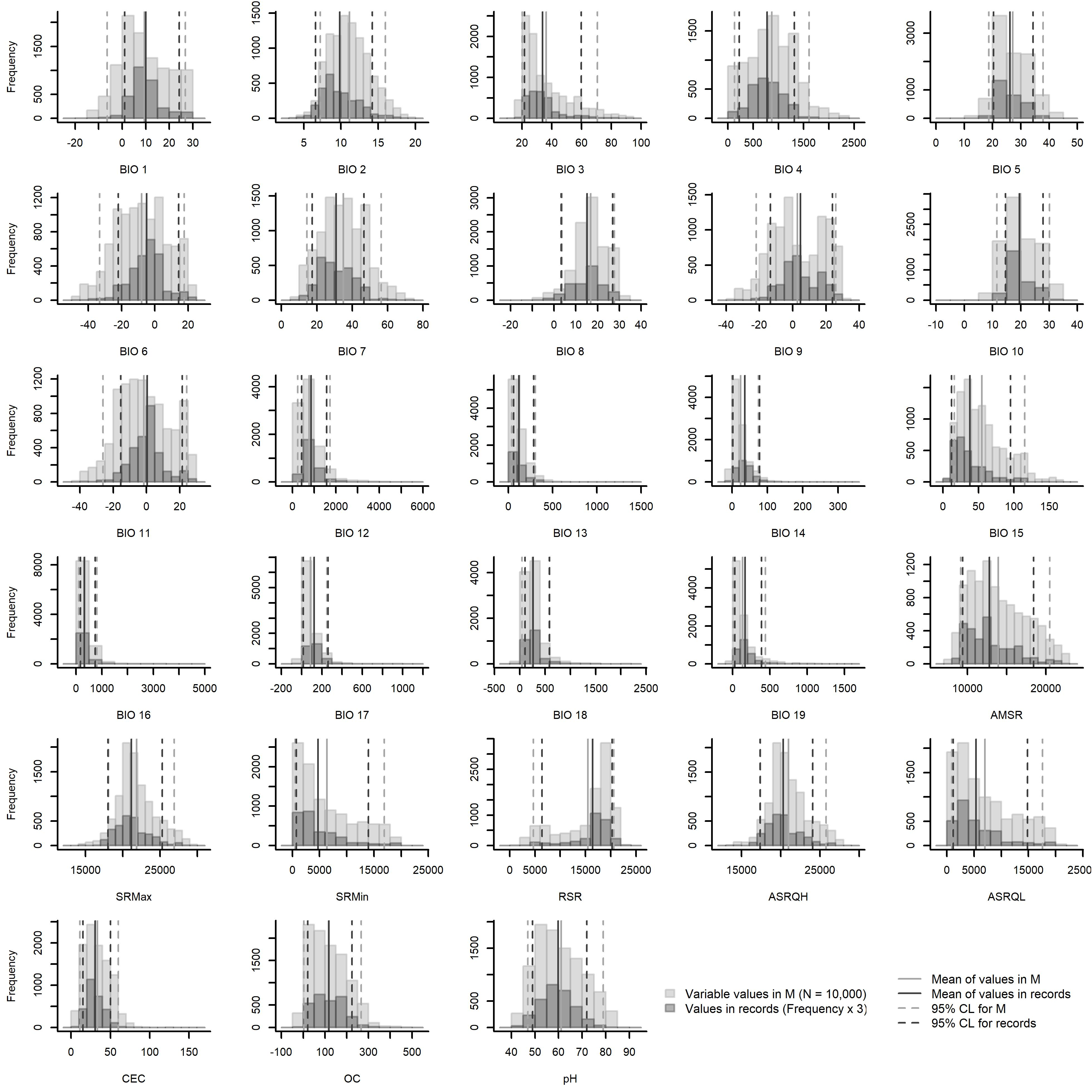

Supplement: S1 Fig — Values of correlation above |0.8| are magnified threefold. Results for variables at 30’ resolution are shown. (TIF) [file pone.0276951.s001.tif]

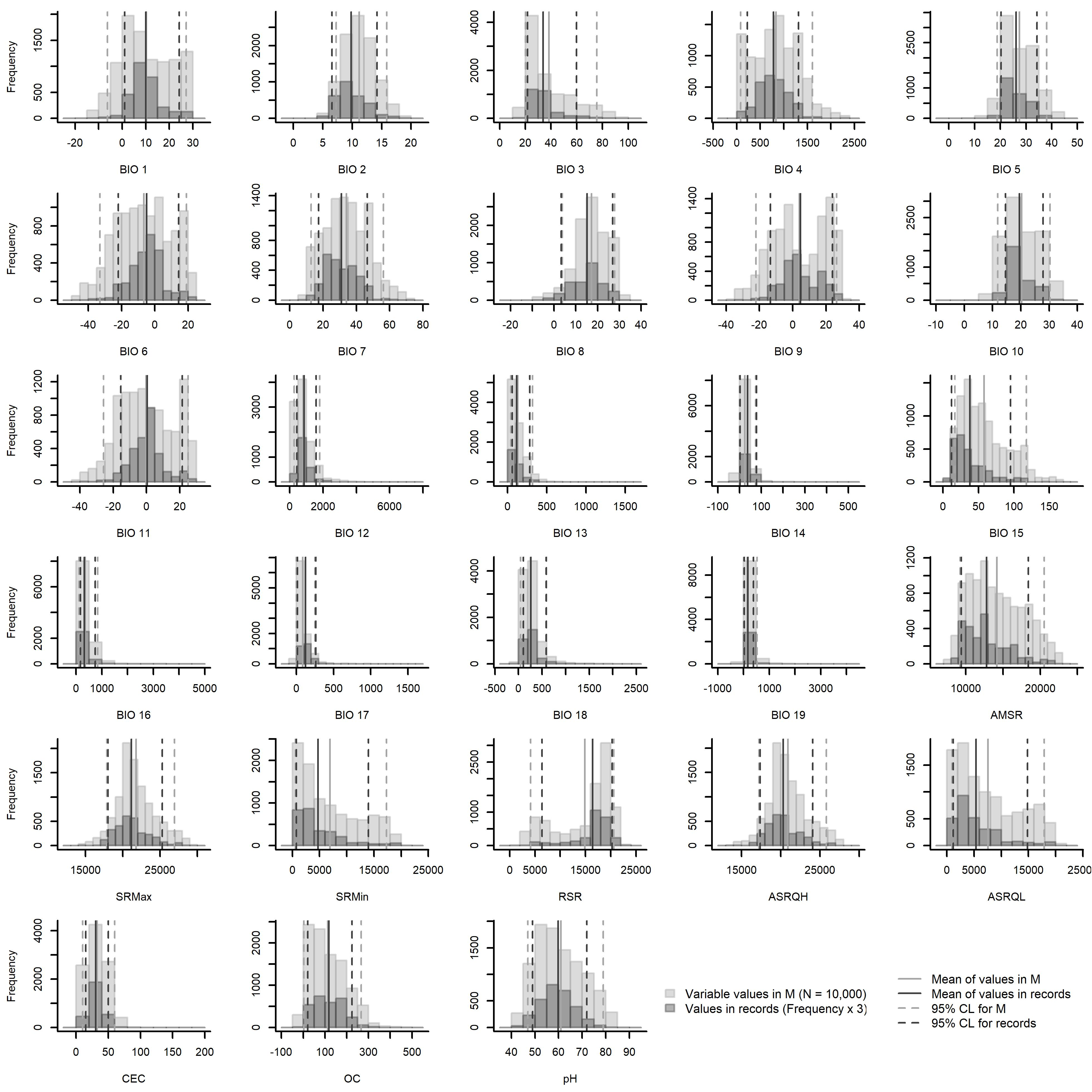

Supplement: S2 Fig — Results for variables at 10’ resolution and buffer calibration areas are shown. (TIF) [file pone.0276951.s002.tif]

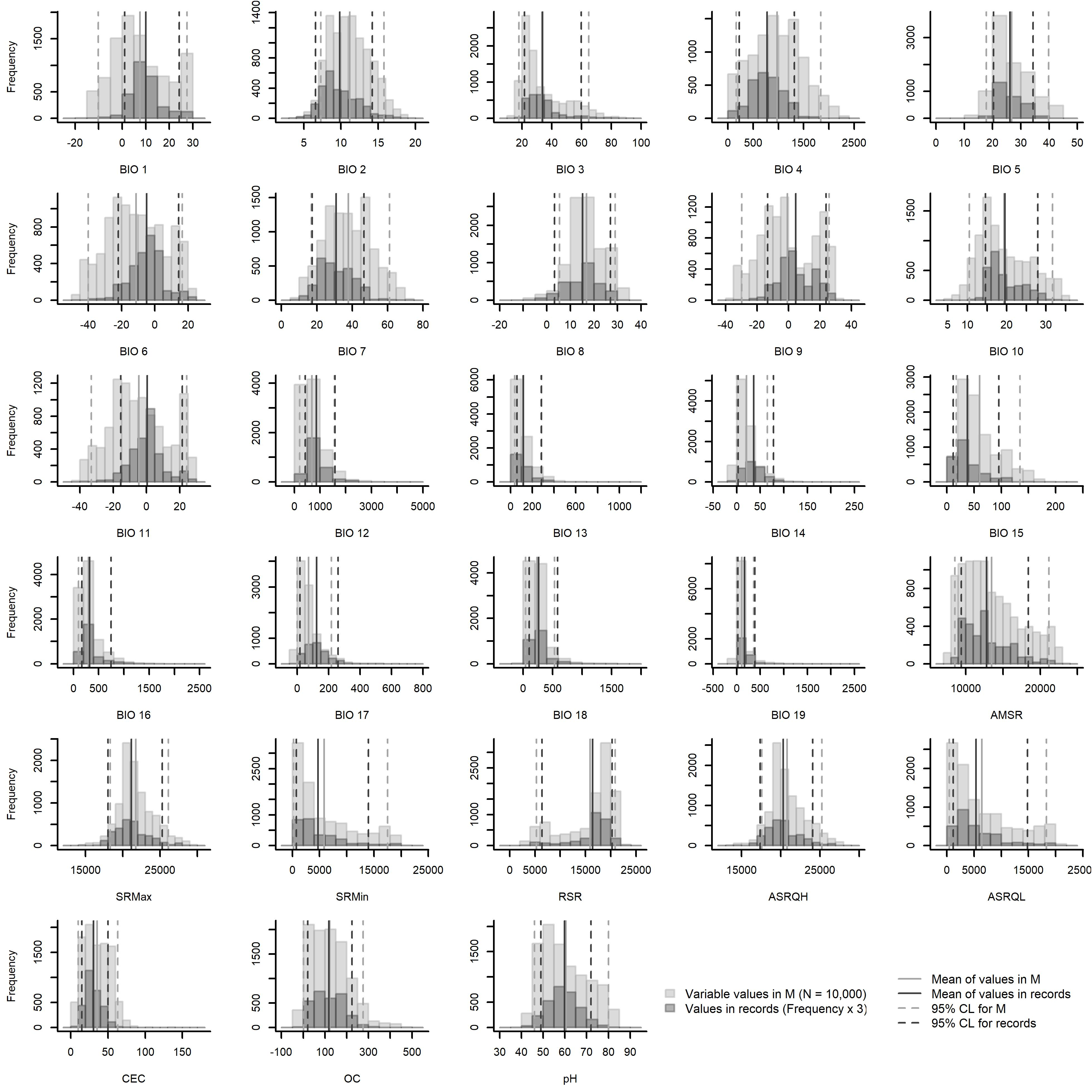

Supplement: S3 Fig — Results for variables at 10’ resolution and concave calibration areas are shown. (TIF) [file pone.0276951.s003.tif]

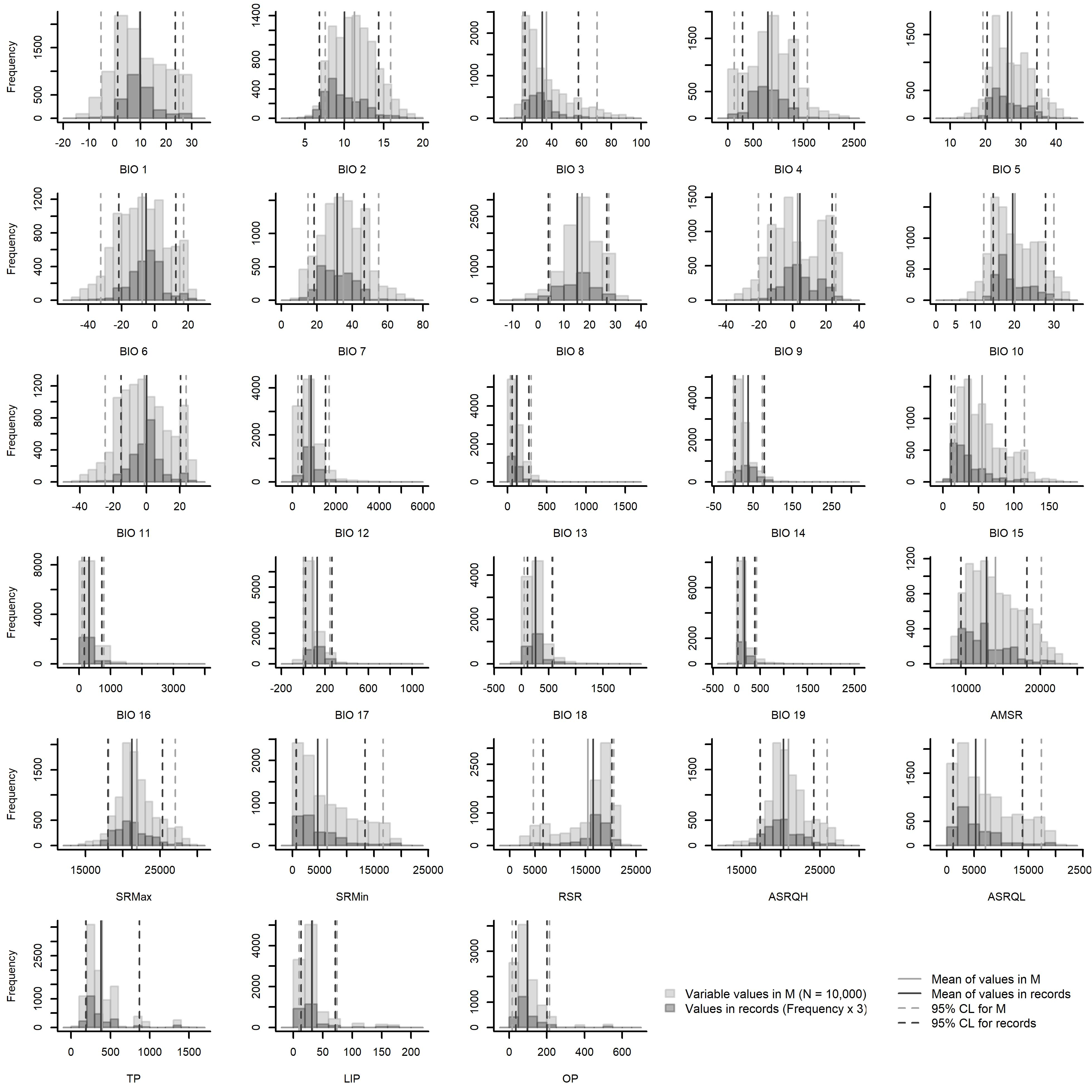

Supplement: S4 Fig — Results for variables at 10’ resolution and calibration areas resulting from ecoregions are shown. (TIF) [file pone.0276951.s004.tif]

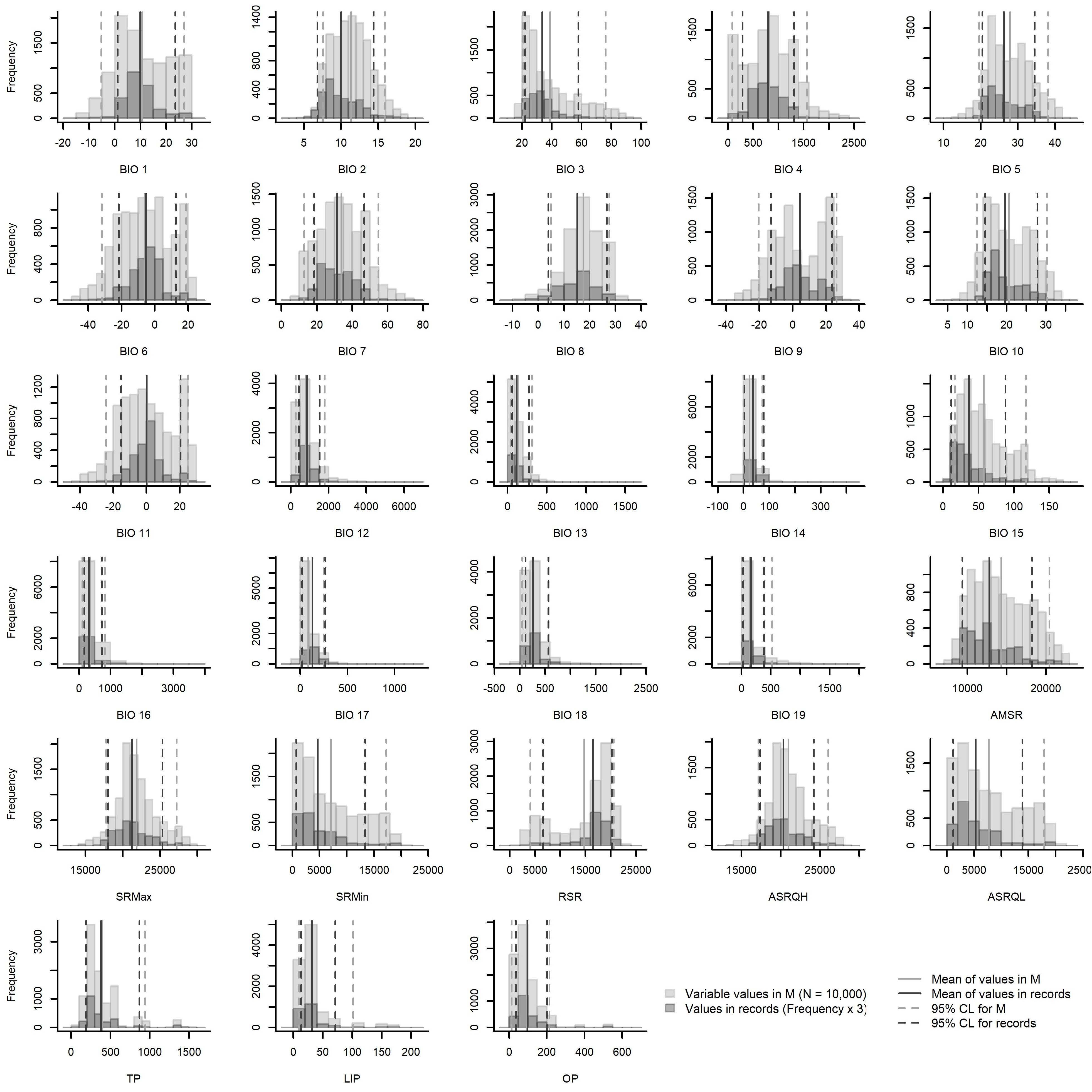

Supplement: S5 Fig — Results for variables at 30’ resolution and buffer calibration areas are shown. (TIF) [file pone.0276951.s005.tif]

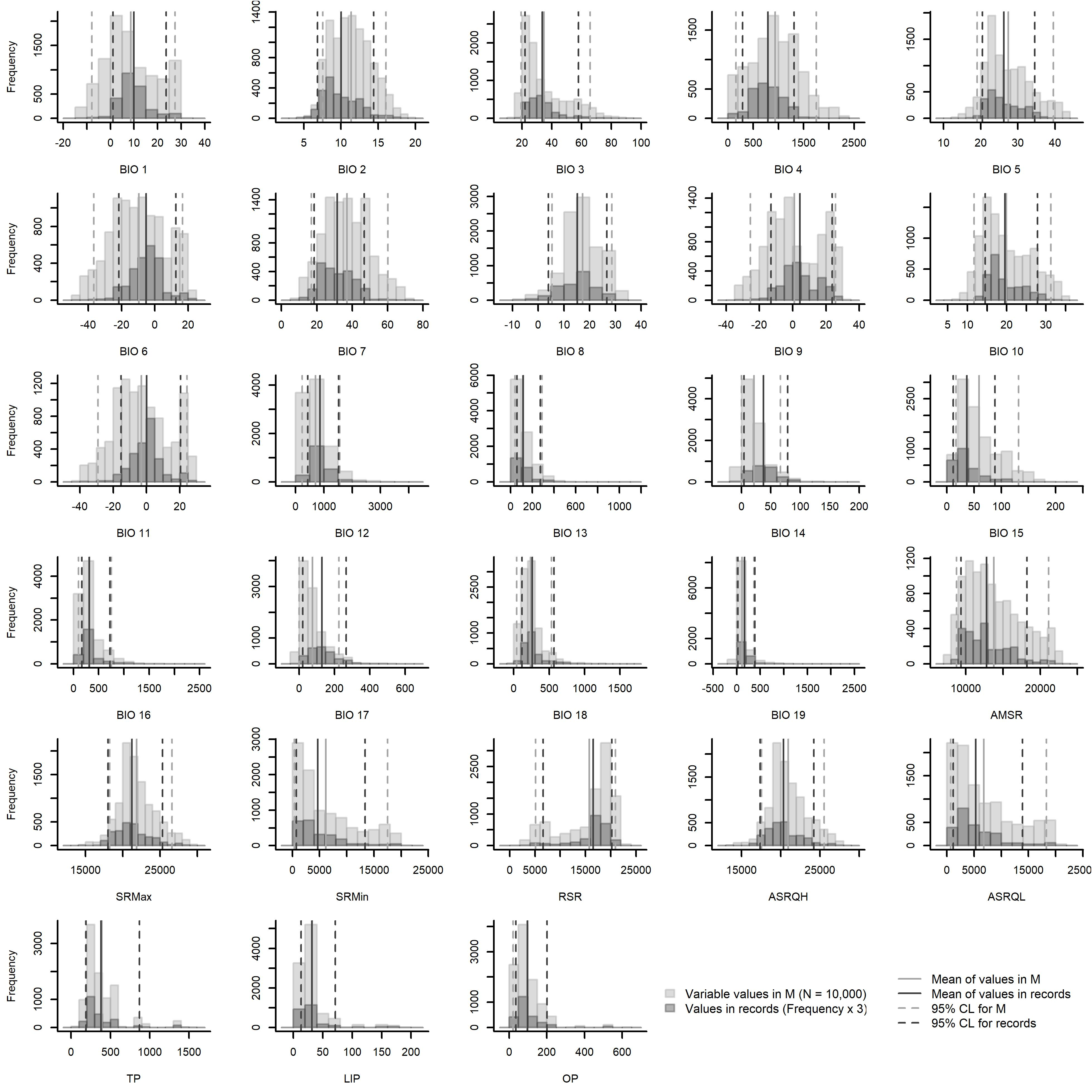

Supplement: S6 Fig — Results for variables at 30’ resolution and concave calibration areas are shown. (TIF) [file pone.0276951.s006.tif]

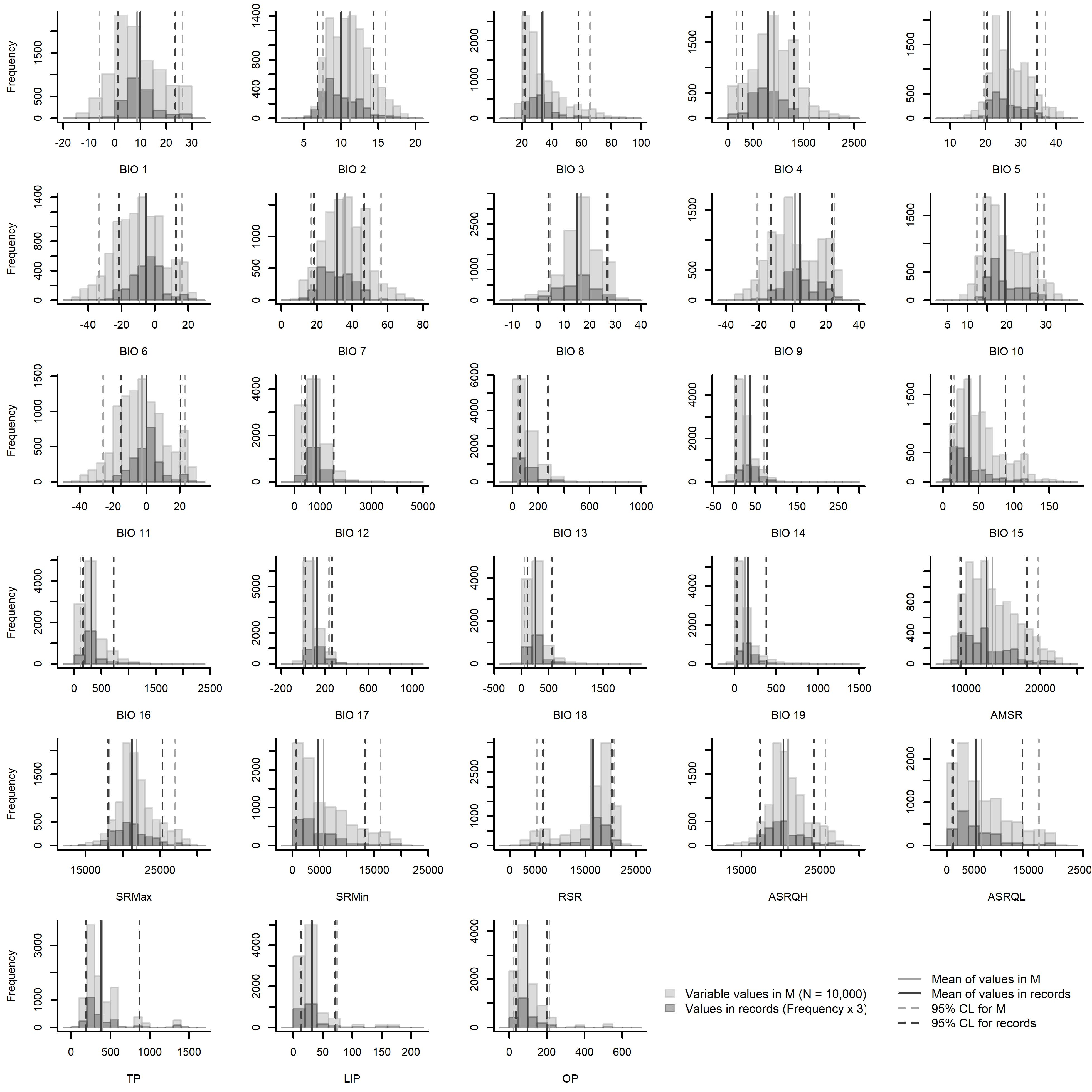

Supplement: S7 Fig — Results for variables at 30’ resolution and calibration areas resulting from ecoregions are shown. (TIF) [file pone.0276951.s007.tif]

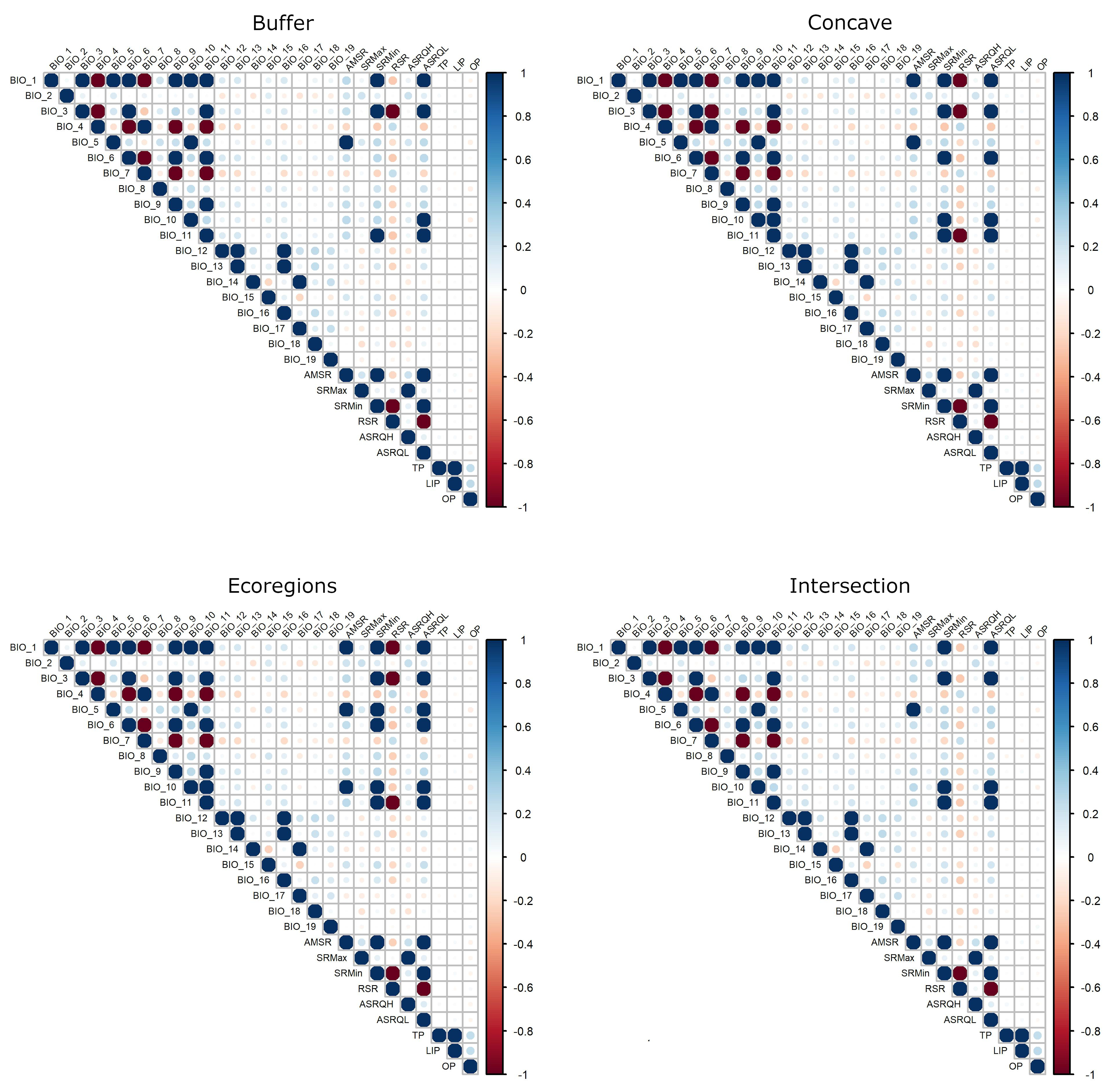

Supplement: S8 Fig — Results for variables at 30’ resolution and calibration areas resulting from intersection are shown. (TIF) [file pone.0276951.s008.tif]

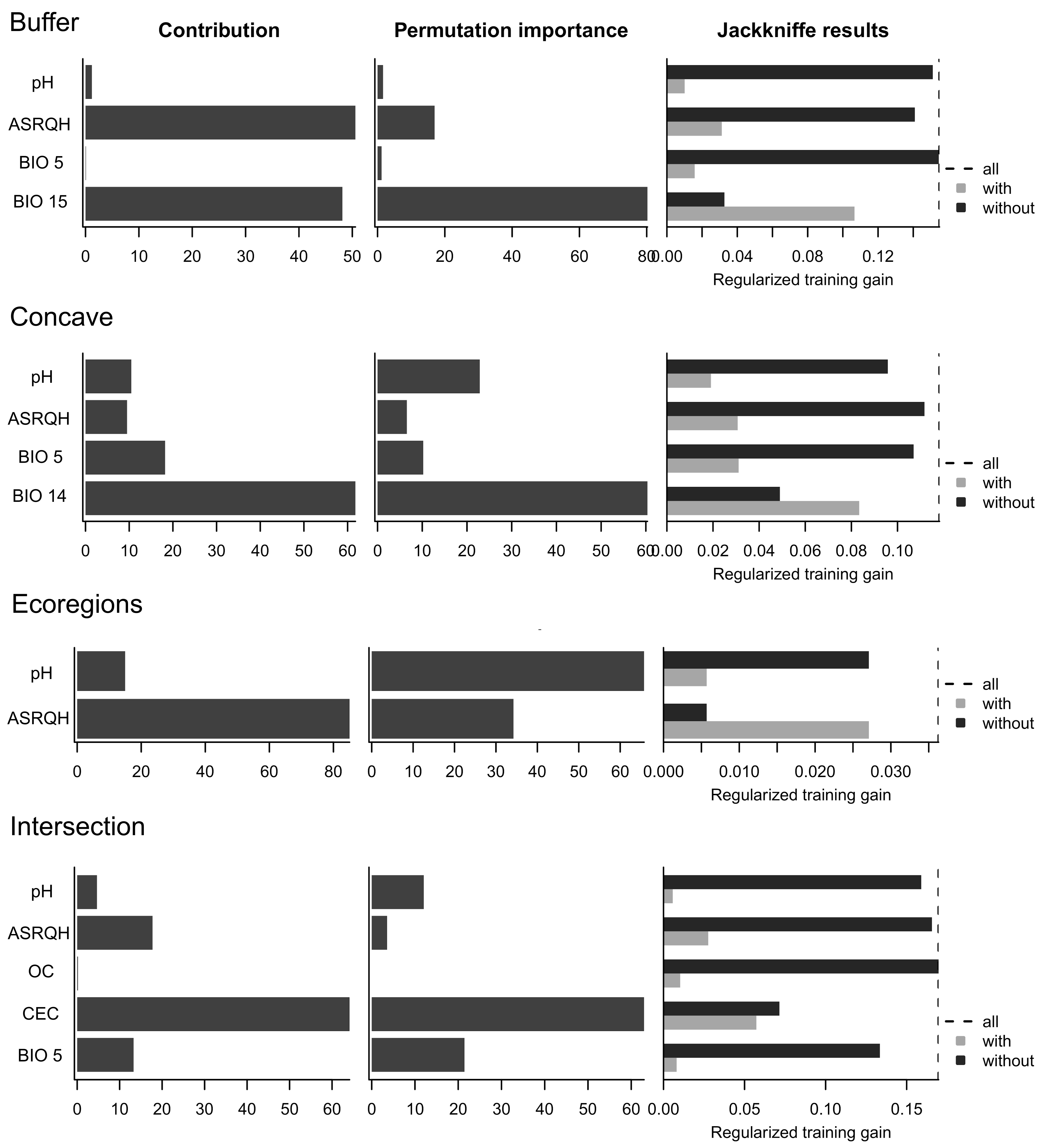

Supplement: S9 Fig — Results for variables at 10’ resolutions are shown. (TIF) [file pone.0276951.s009.tif]

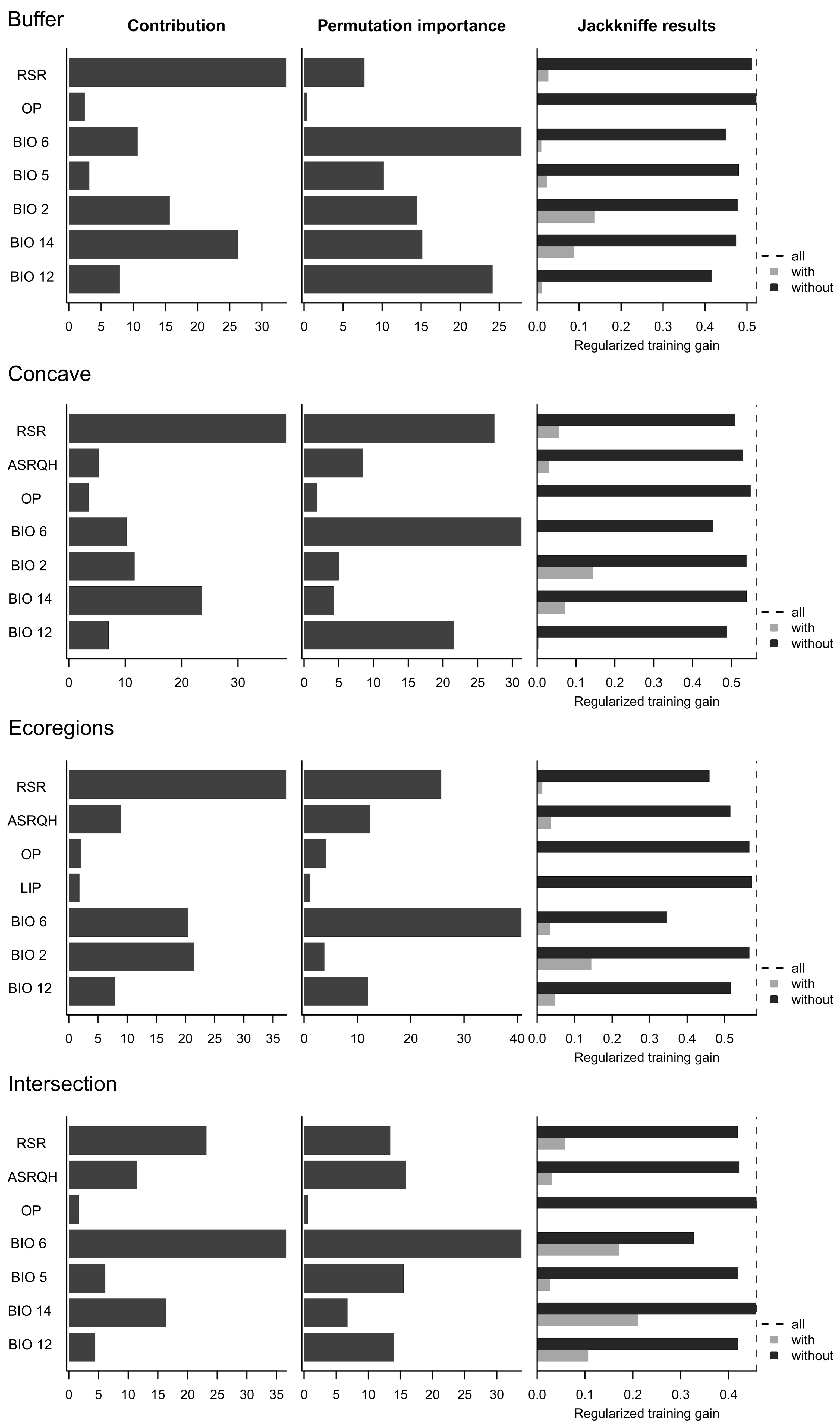

Supplement: S10 Fig — Results for variables at 30’ resolutions are shown. (TIF) [file pone.0276951.s010.tif]

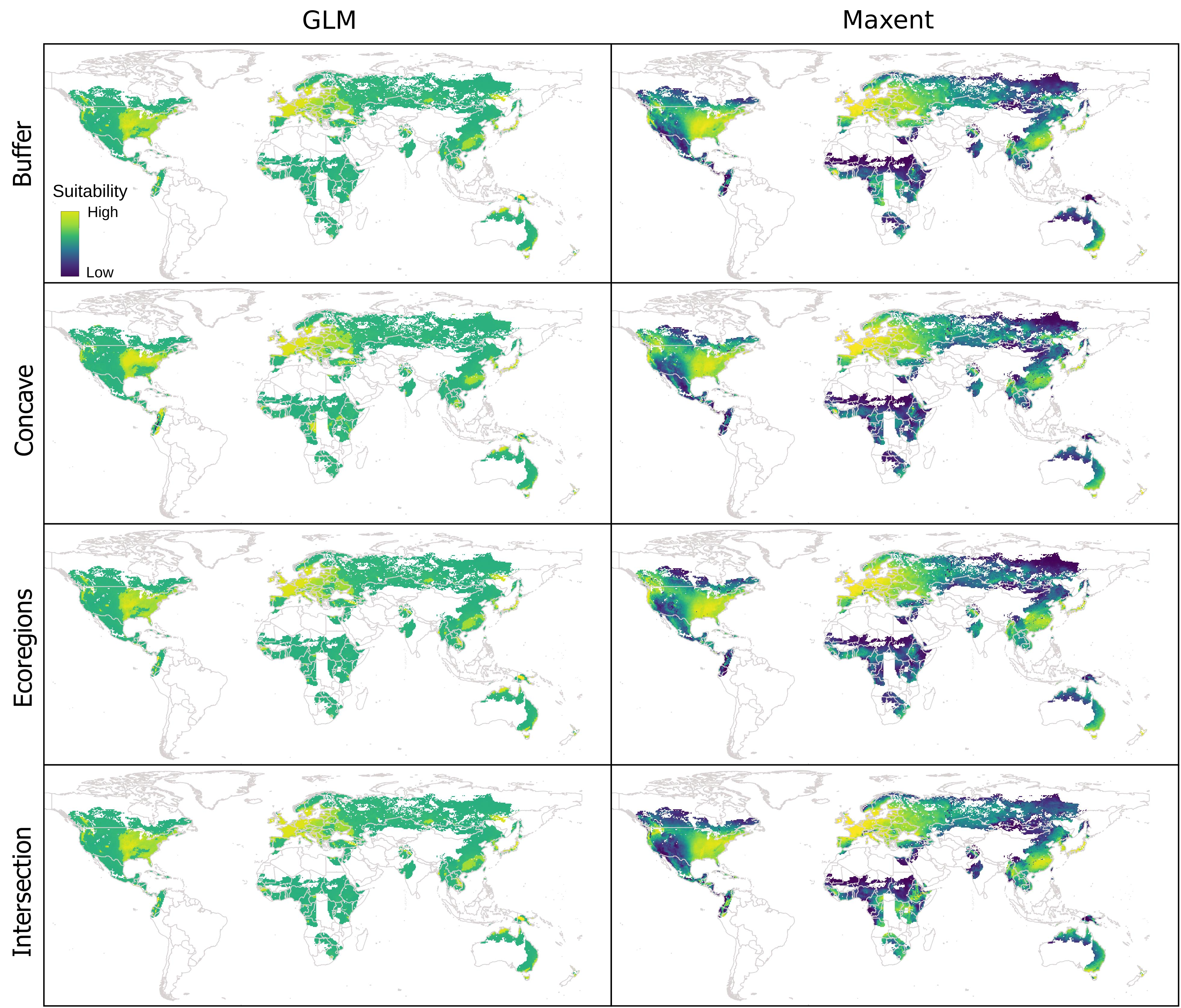

Supplement: S11 Fig — Results for variables at 30’ resolution are shown. (TIF) [file pone.0276951.s011.tif]

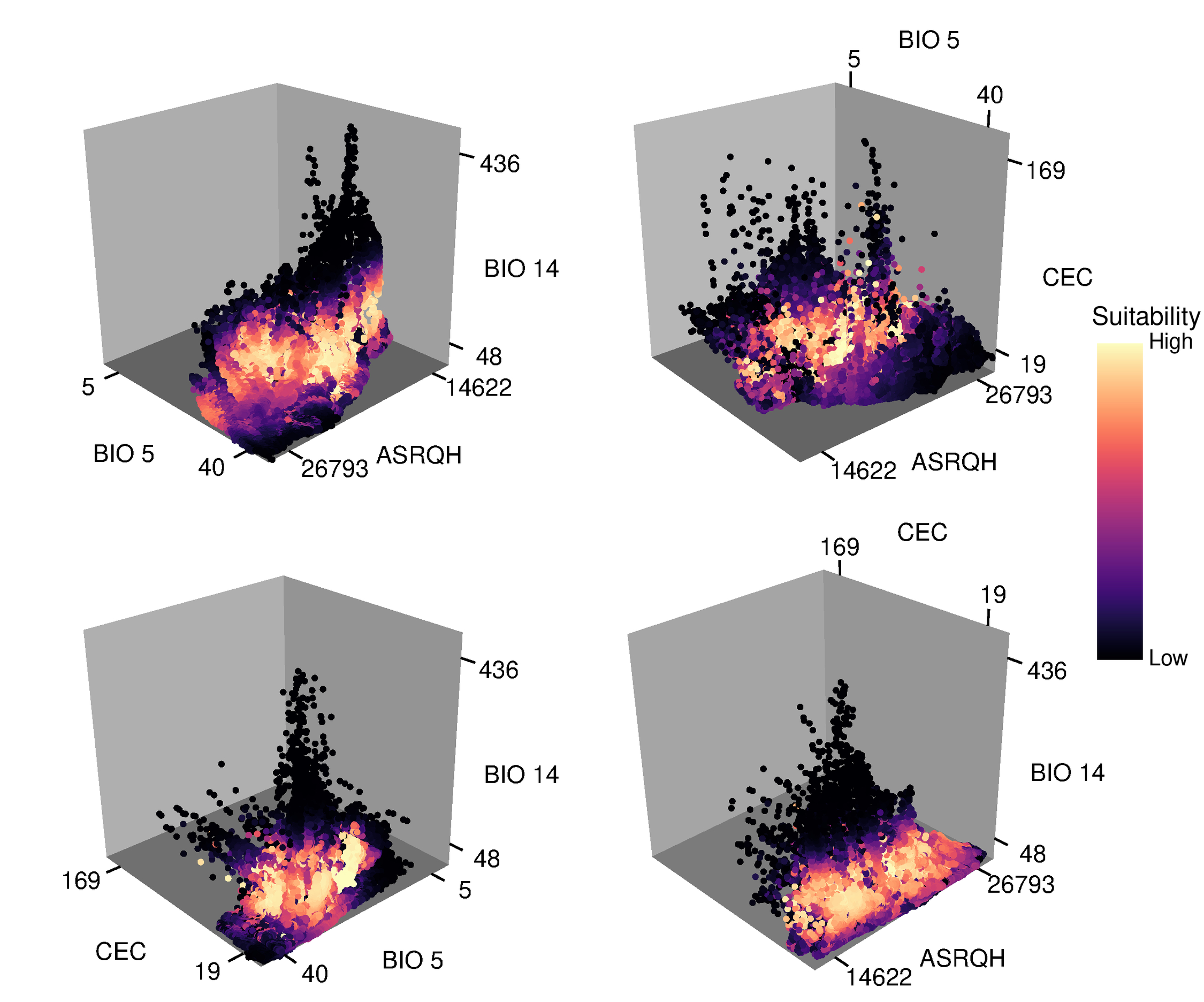

Supplement: S12 Fig — Values of suitability derive from final models created with selected variables and parameters. GLM results for variables at 10’ resolution and calibration areas resulting from buffers are shown. (TIF) [file pone.0276951.s012.tif]

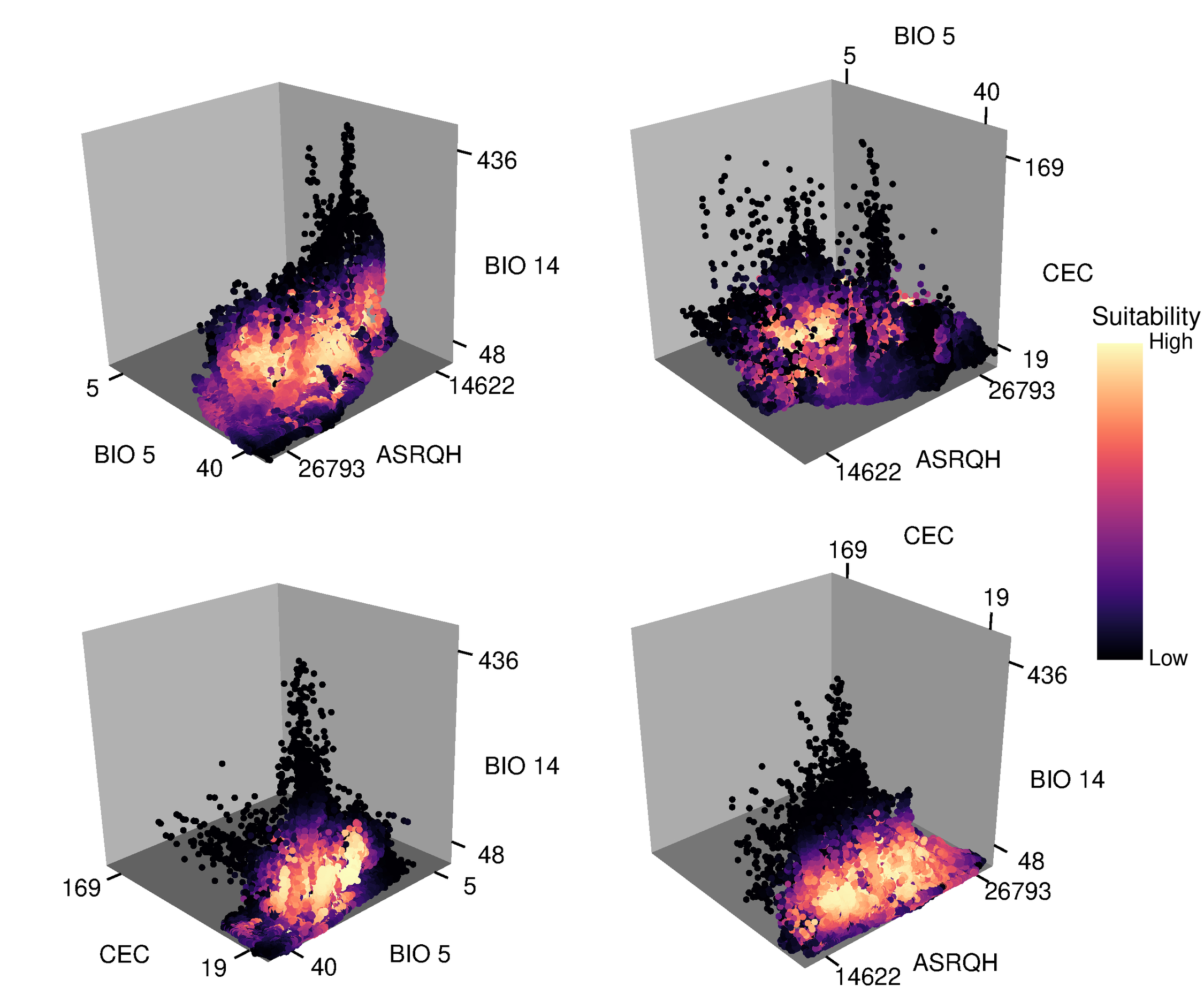

Supplement: S13 Fig — Values of suitability derive from final models created with selected variables and parameters. GLM results for variables at 10’ resolution and calibration areas resulting from concave hulls are shown. (TIF) [file pone.0276951.s013.tif]

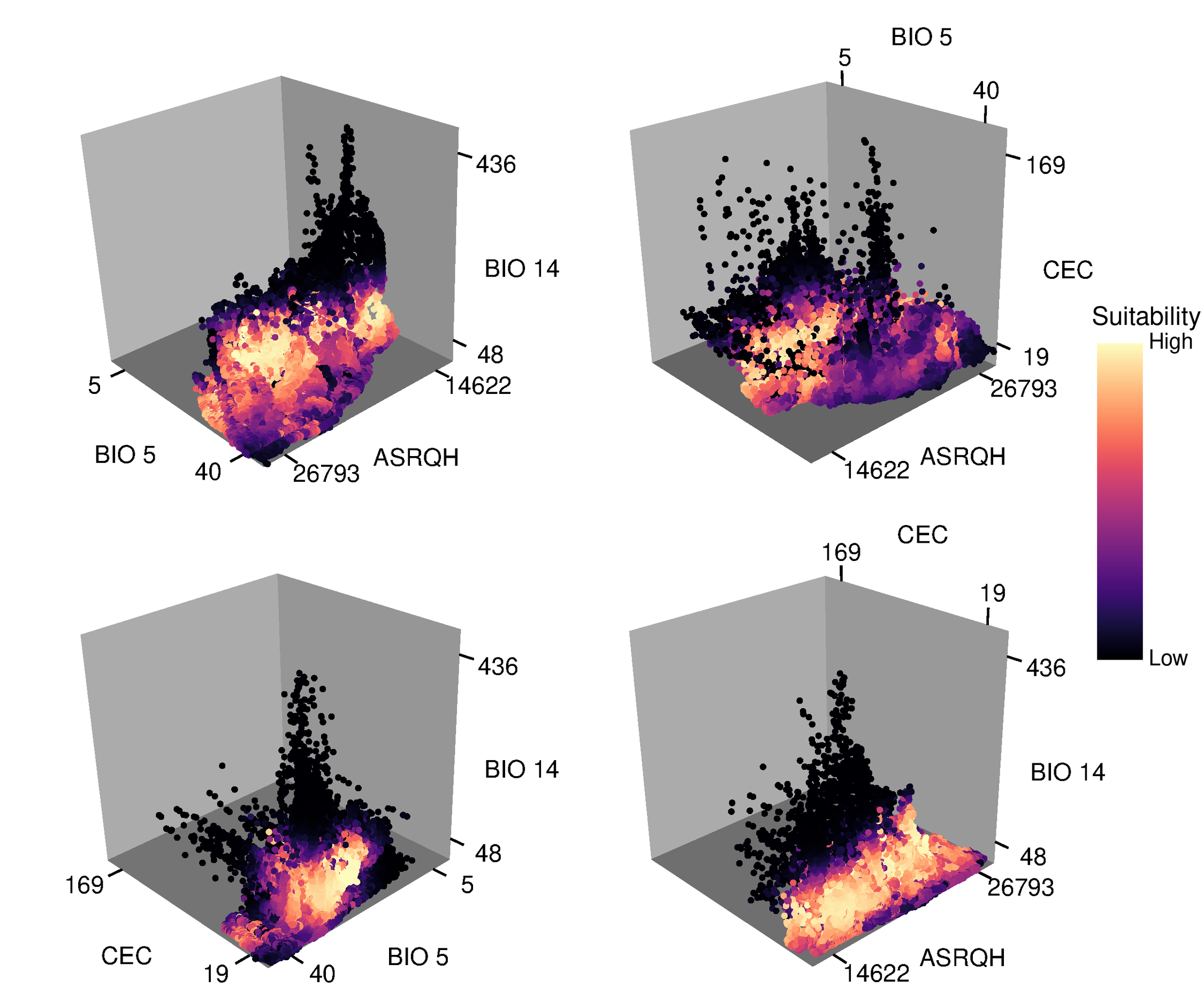

Supplement: S14 Fig — Values of suitability derive from final models created with selected variables and parameters. GLM results for variables at 10’ resolution and calibration areas resulting from ecoregions are shown. (TIF) [file pone.0276951.s014.tif]

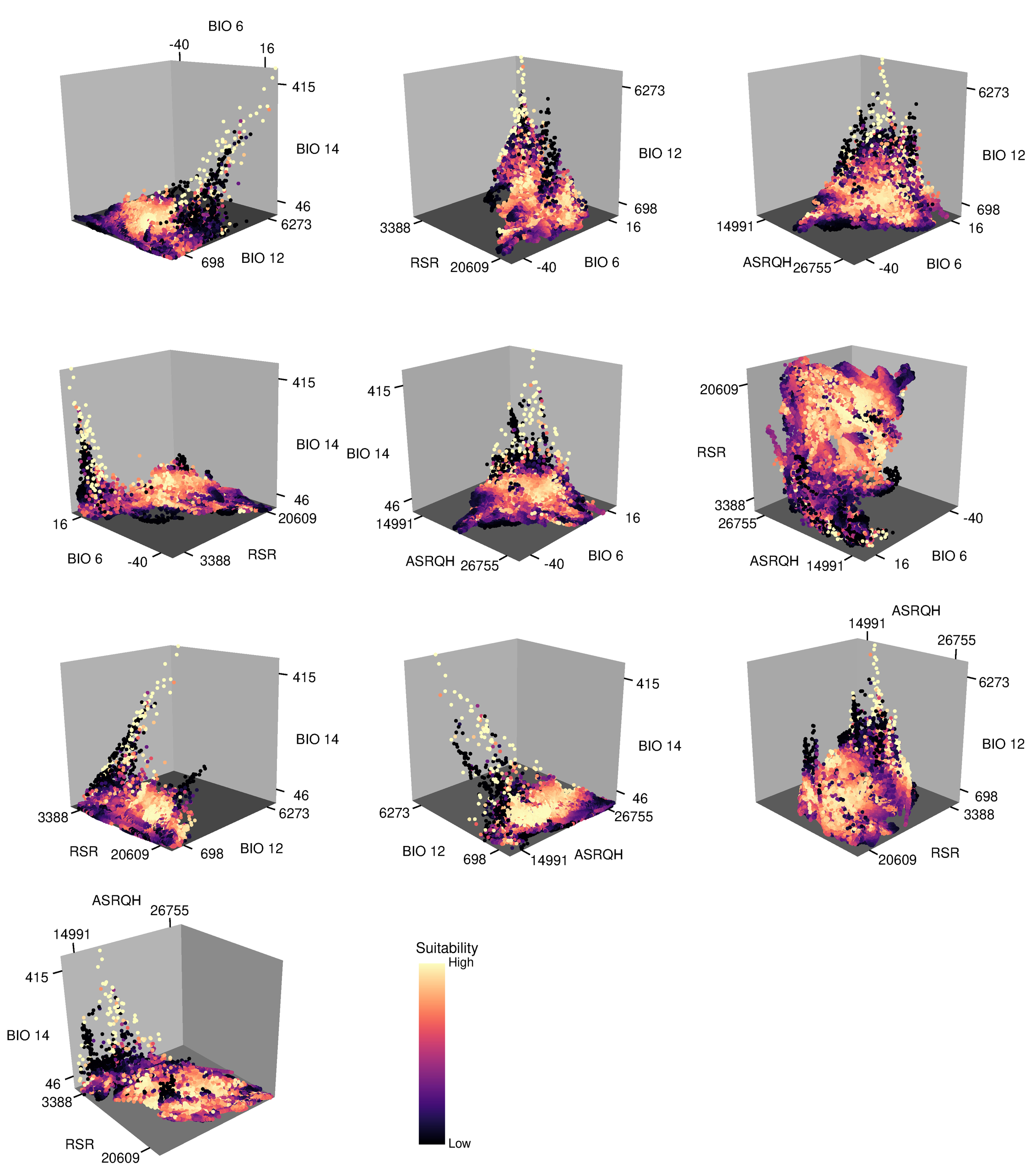

Supplement: S15 Fig — Values of suitability derive from final models created with selected variables and parameters. GLM results for variables at 30’ resolution and calibration areas resulting from buffers are shown. (TIF) [file pone.0276951.s015.tif]

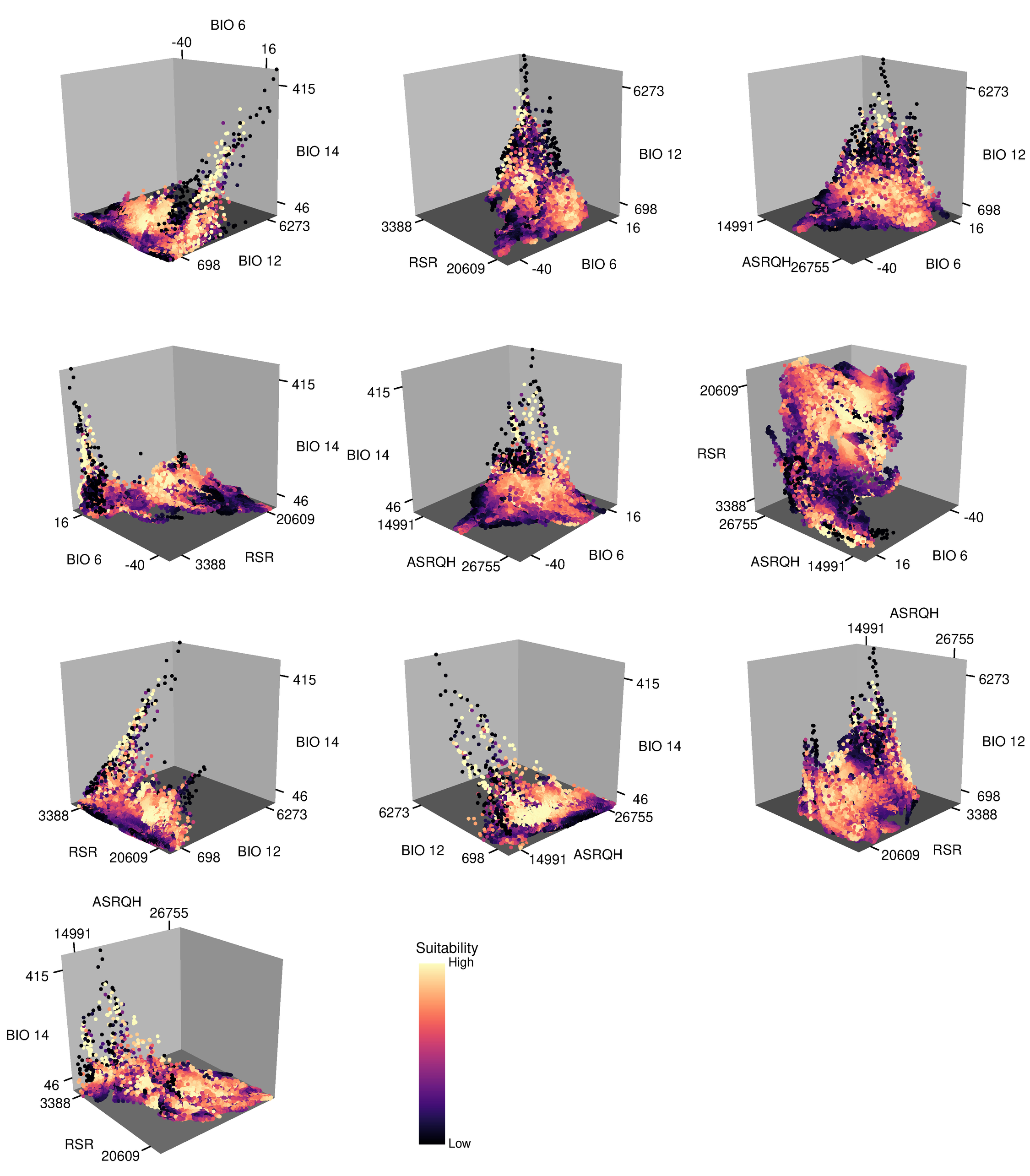

Supplement: S16 Fig — Values of suitability derive from final models created with selected variables and parameters. GLM results for variables at 30’ resolution and calibration areas resulting from concave hulls are shown. (TIF) [file pone.0276951.s016.tif]

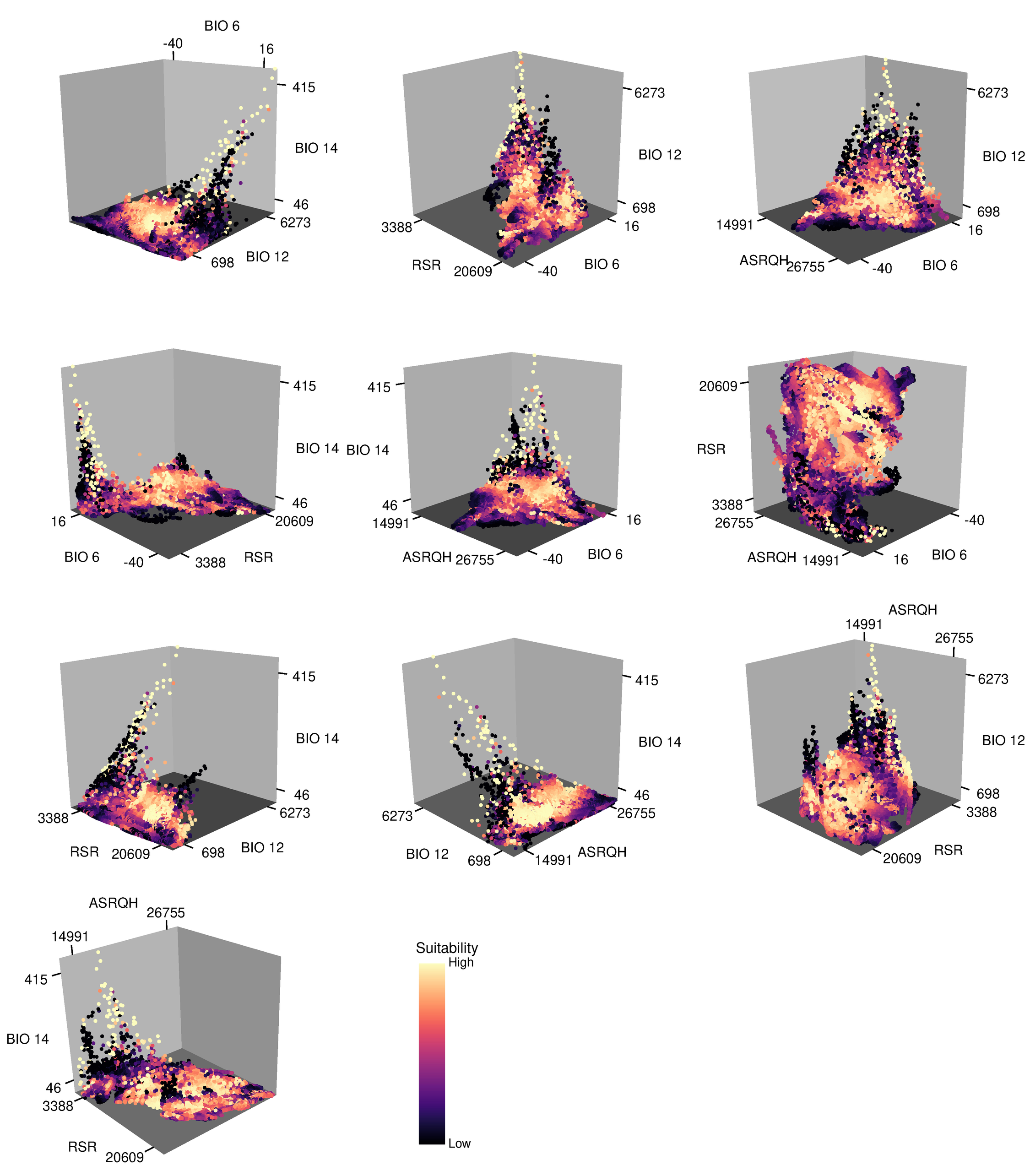

Supplement: S17 Fig — Values of suitability derive from final models created with selected variables and parameters. GLM results for variables at 30’ resolution and calibration areas resulting from ecoregions are shown. (TIF) [file pone.0276951.s017.tif]

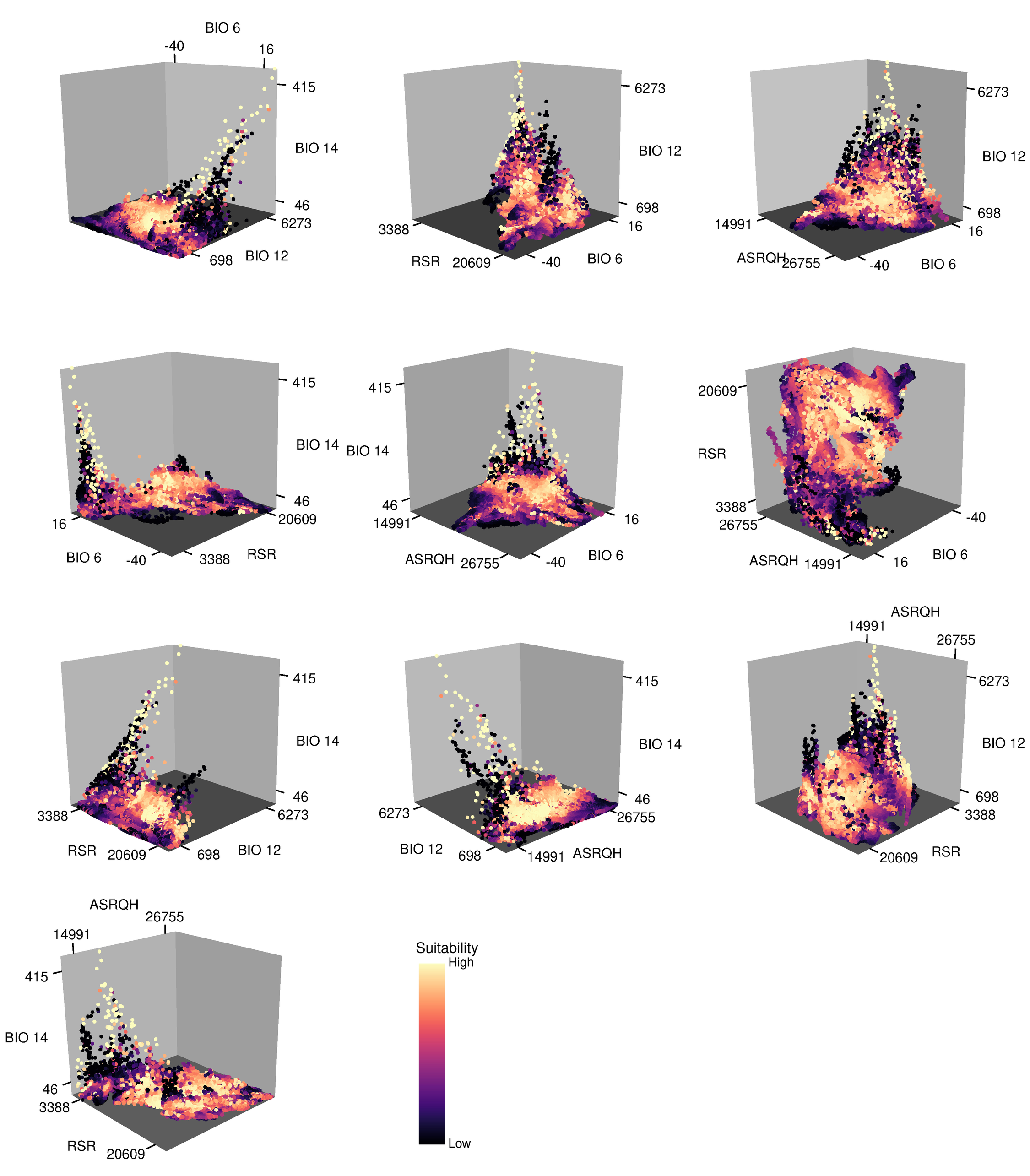

Supplement: S18 Fig — Values of suitability derive from final models created with selected variables and parameters. GLM results for variables at 30’ resolution and calibration areas resulting from intersection are shown. (TIF) [file pone.0276951.s018.tif]

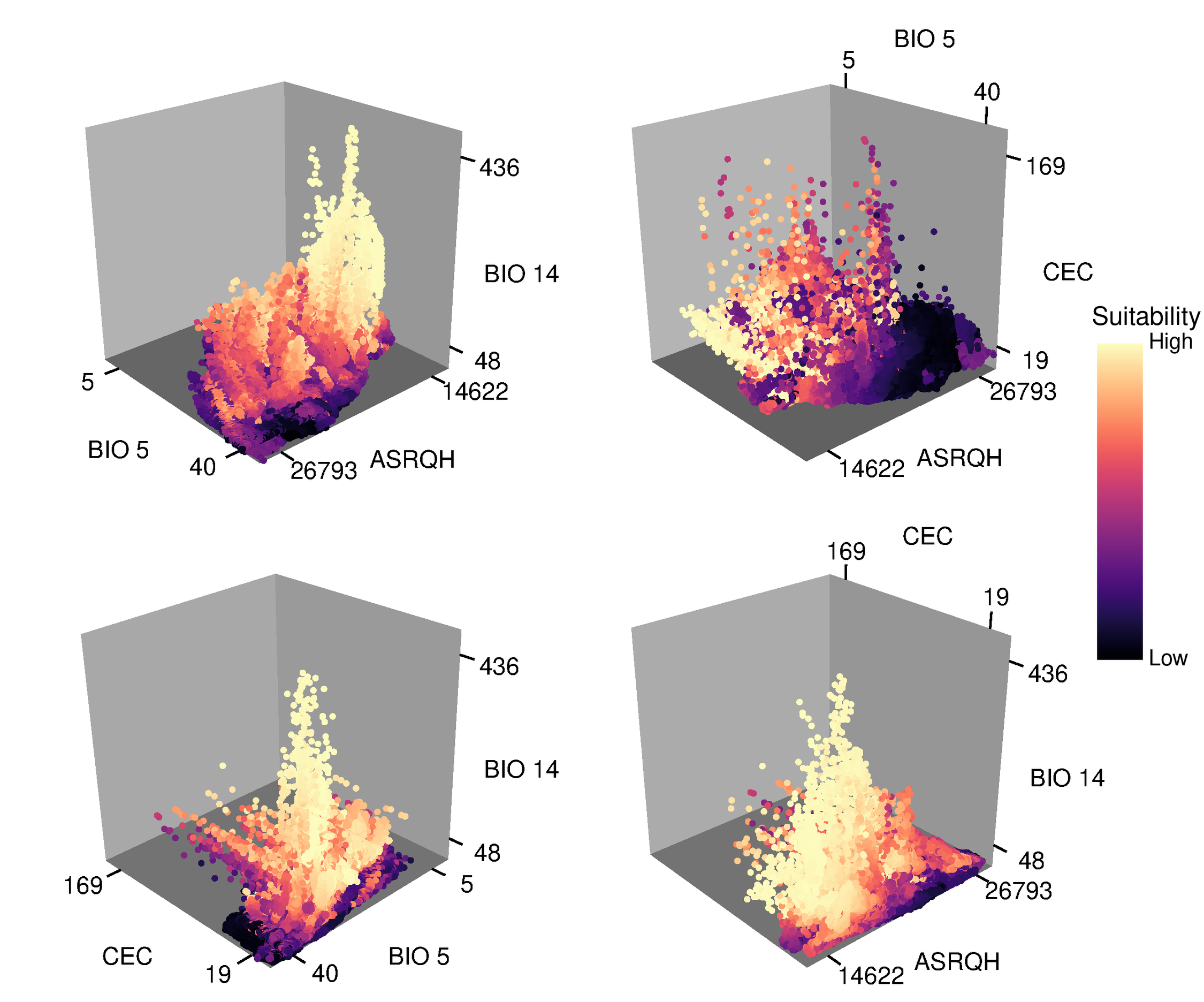

Supplement: S19 Fig — Values of suitability derive from final models created with selected variables and parameters. Maxent results for variables at 10’ resolution and calibration areas resulting from buffers are shown. (TIF) [file pone.0276951.s019.tif]

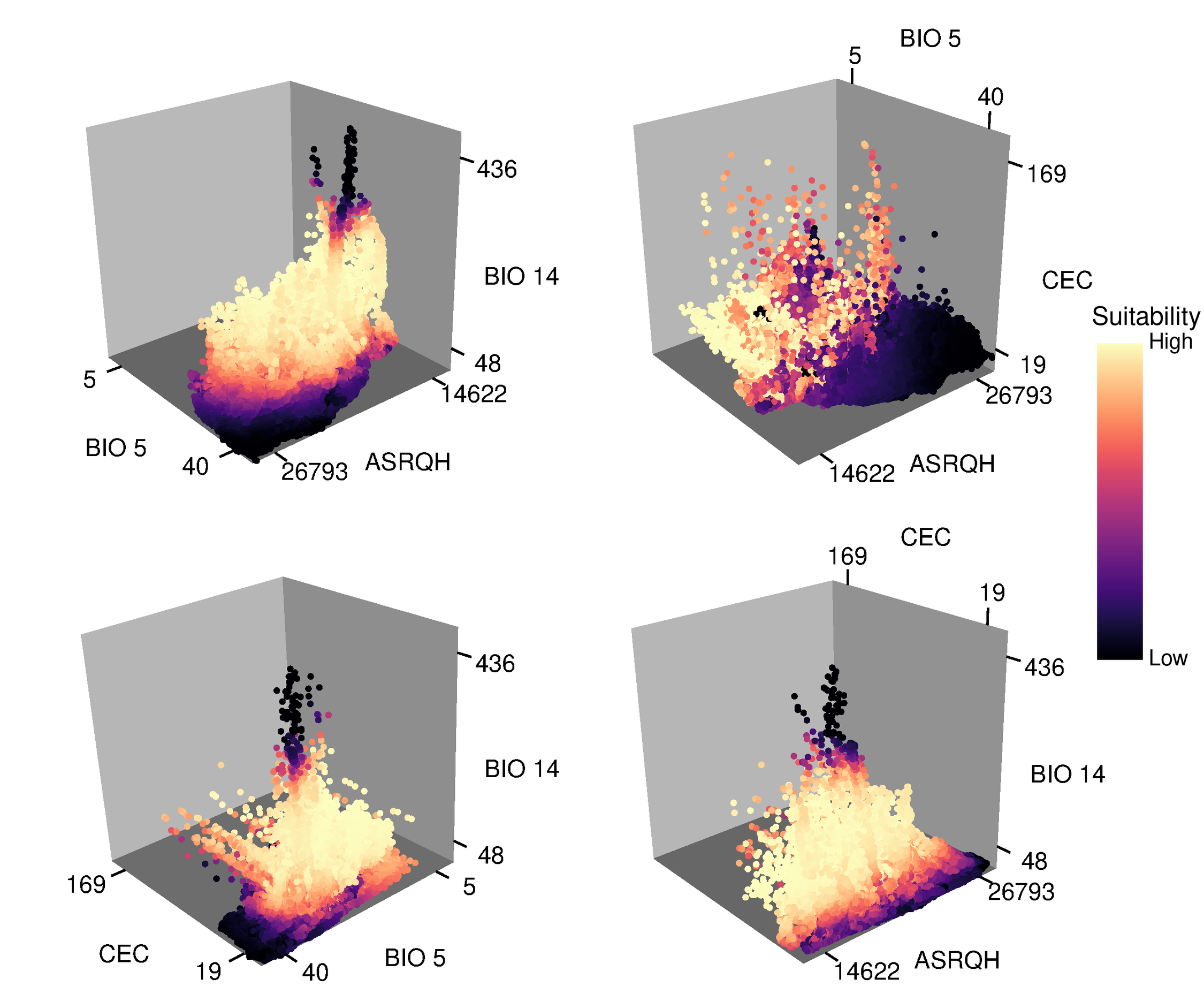

Supplement: S20 Fig — Values of suitability derive from final models created with selected variables and parameters. Maxent results for variables at 10’ resolution and calibration areas resulting from concave hulls are shown. (TIF) [file pone.0276951.s020.tif]

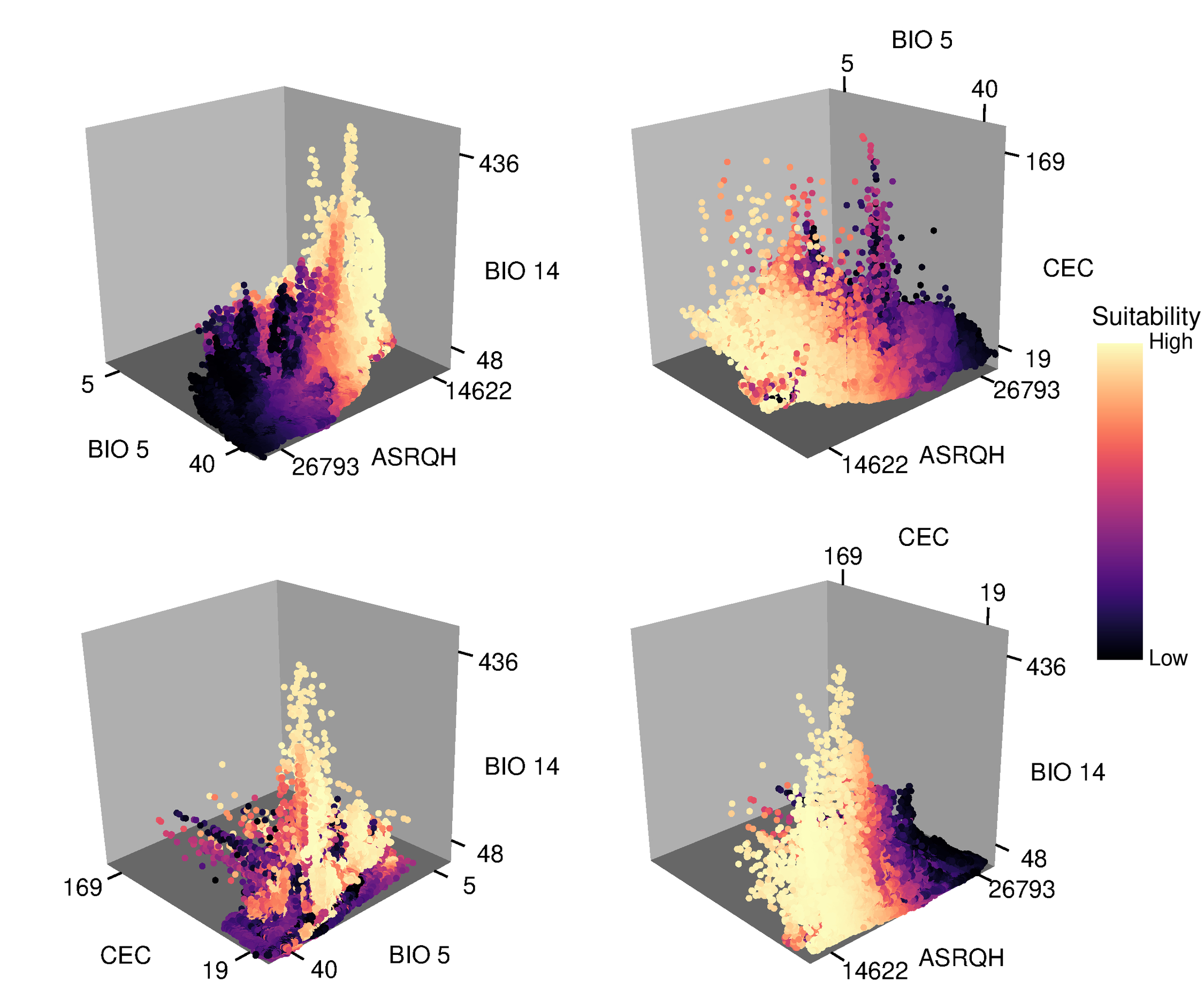

Supplement: S21 Fig — Values of suitability derive from final models created with selected variables and parameters. Maxent results for variables at 10’ resolution and calibration areas resulting from ecoregions are shown. (TIF) [file pone.0276951.s021.tif]

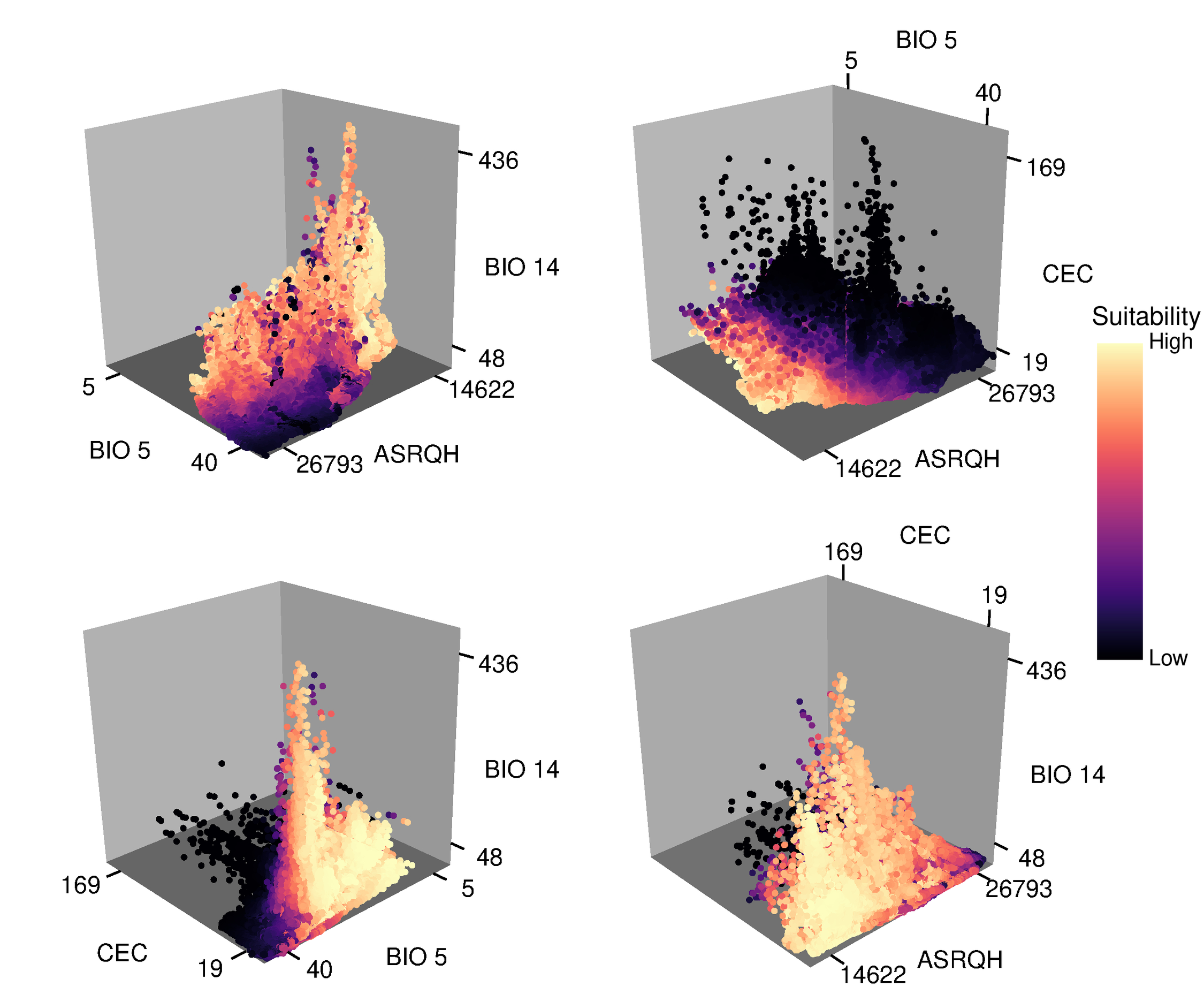

Supplement: S22 Fig — Values of suitability derive from final models created with selected variables and parameters. Maxent results for variables at 10’ resolution and calibration areas resulting from intersection are shown. (TIF) [file pone.0276951.s022.tif]

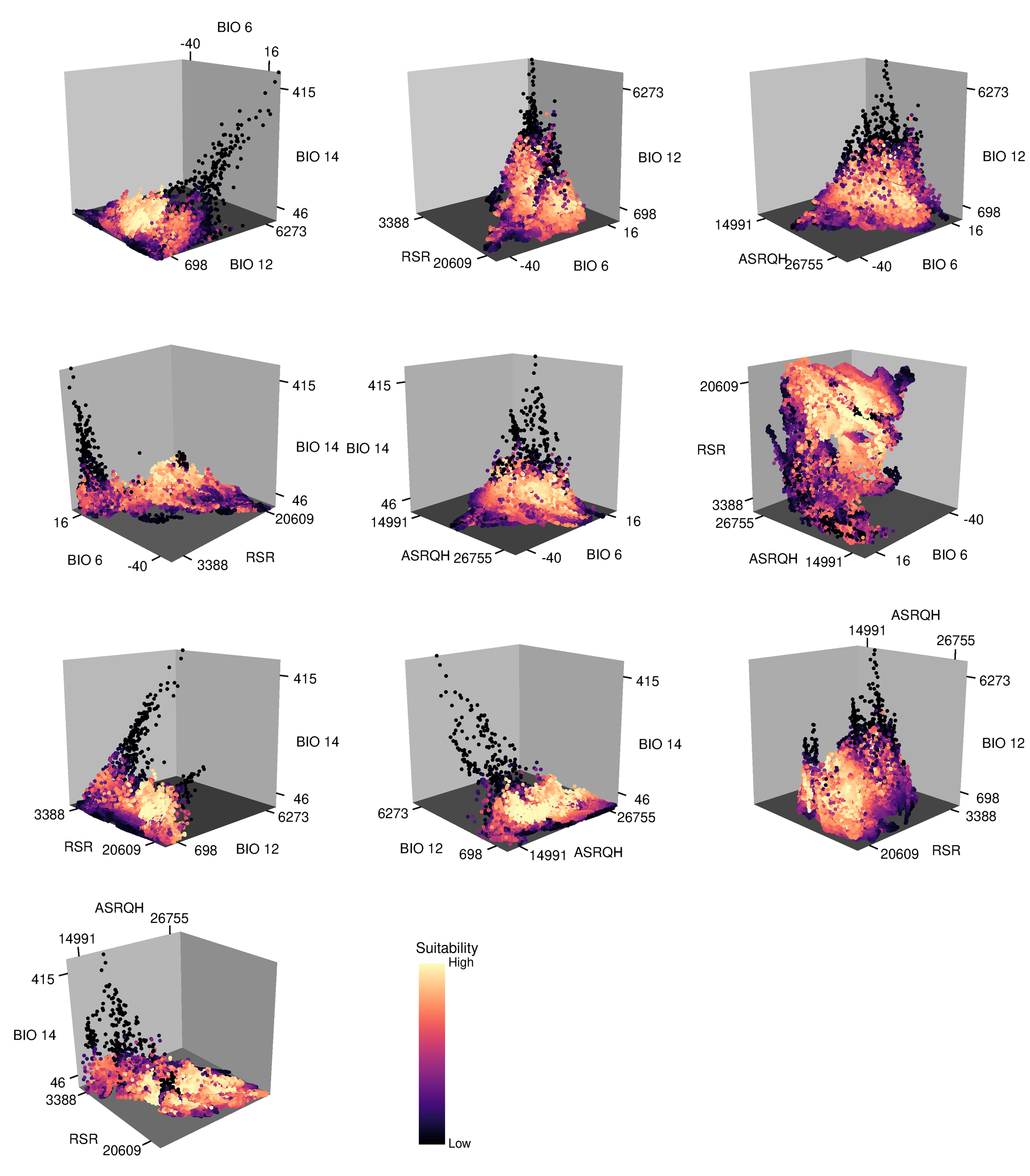

Supplement: S23 Fig — Values of suitability derive from final models created with selected variables and parameters. Maxent results for variables at 30’ resolution and calibration areas resulting from buffers are shown. (TIF) [file pone.0276951.s023.tif]

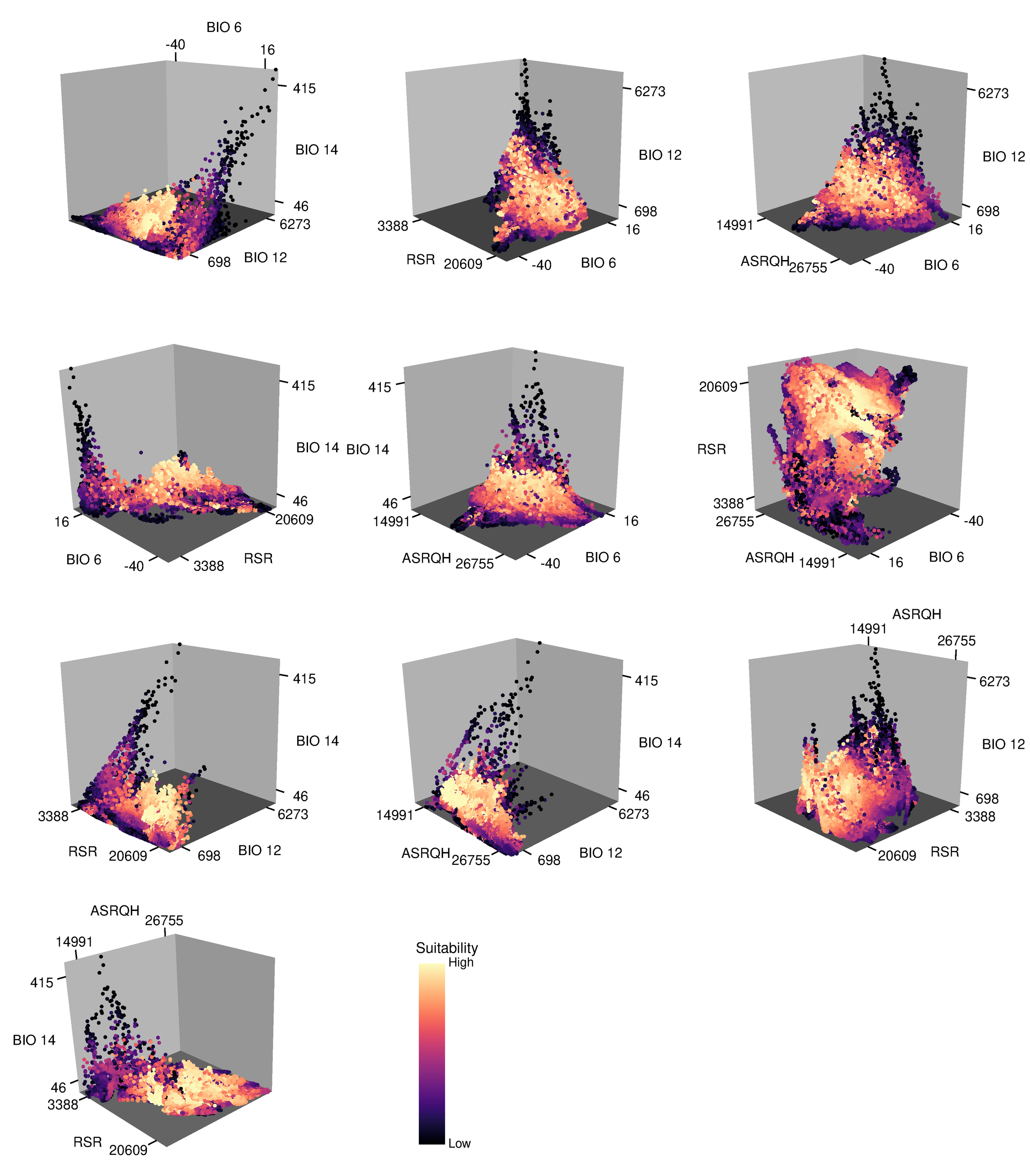

Supplement: S24 Fig — Values of suitability derive from final models created with selected variables and parameters. Maxent results for variables at 30’ resolution and calibration areas resulting from concave hulls are shown. (TIF) [file pone.0276951.s024.tif]

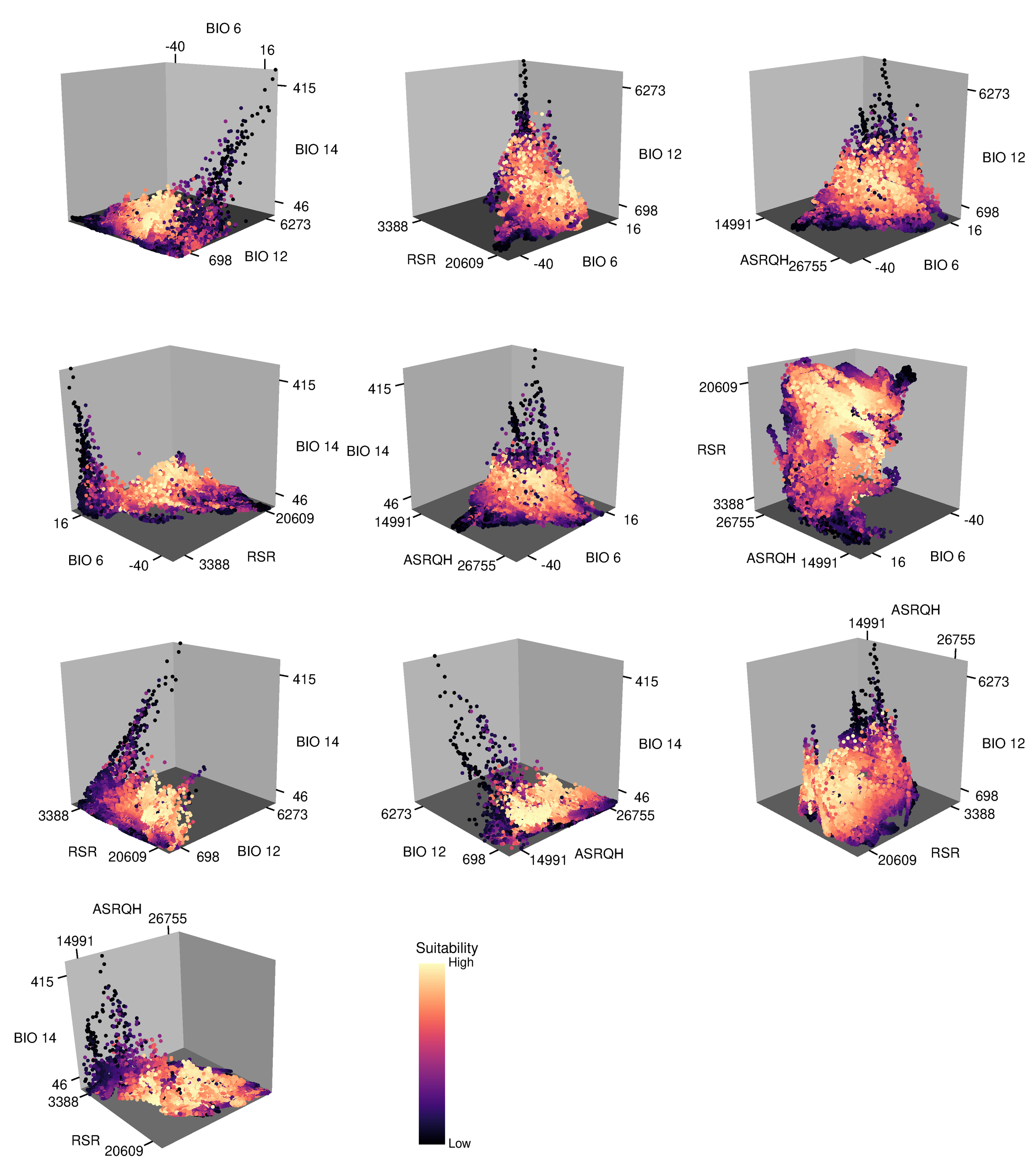

Supplement: S25 Fig — Values of suitability derive from final models created with selected variables and parameters. Maxent results for variables at 30’ resolution and calibration areas resulting from ecoregions are shown. (TIF) [file pone.0276951.s025.tif]

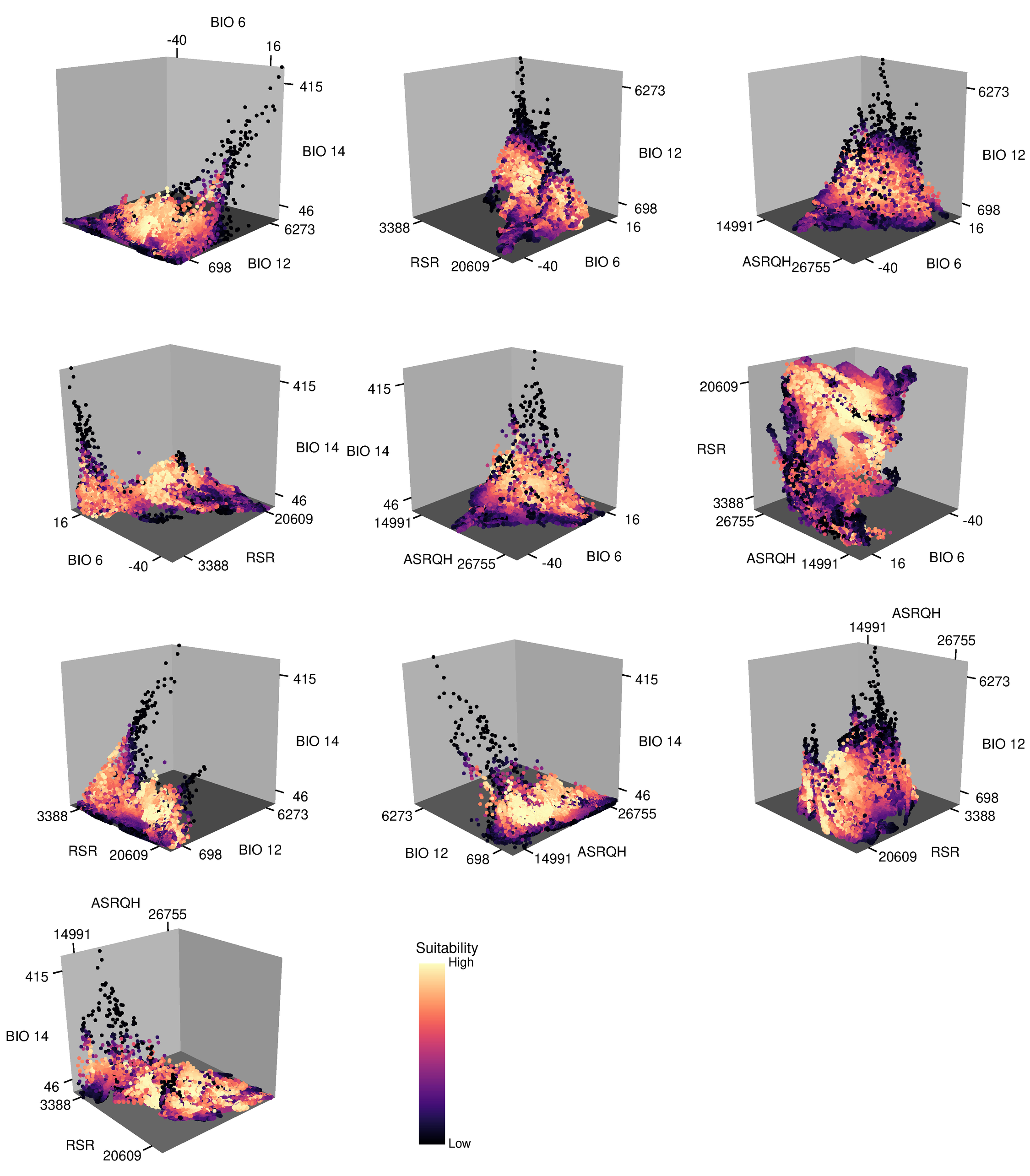

Supplement: S26 Fig — Values of suitability derive from final models created with selected variables and parameters. Maxent results for variables at 30’ resolution and calibration areas resulting from intersection are shown. (TIF) [file pone.0276951.s026.tif]
