# Supplementary material for: Broad-scale factors shaping the ecological niche and geographic distribution of Spirodela polyrhiza
Source: PLoS One. 2023 May 4;18(5):e0276951. doi: 10.1371/journal.pone.0276951 (PMC10159170; doi:10.1371/journal.pone.0276951)
Supplement: S2 Table — Results presented here are for variables at 30’ resolution. Spatial autocorrelation was measured using the statistic Moran’s I. (DOCX) [file pone.0276951.s028.docx]

S2 Table. Spatial autocorrelation results for all environmental variables derived from spatial patterns of occurrence data after using distinct distances for spatial thinning. Results presented here are for variables at 30’ resolution. Spatial autocorrelation was measured using the statistic Moran’s I.

| Remaining points | Thinning distance (km) | Variables | Observed (I) | Expected (I) | SD (I) | *P* value |
| --- | --- | --- | --- | --- | --- | --- |
| 45583 | 0 | BIO 2 | 13.282 | 0.000 | 0.177 | 0.000 |
| 45583 | 0 | BIO 5 | 51.914 | 0.000 | 0.177 | 0.000 |
| 45583 | 0 | BIO 6 | 26.103 | 0.000 | 0.177 | 0.000 |
| 45583 | 0 | BIO 12 | 24.763 | 0.000 | 0.177 | 0.000 |
| 45583 | 0 | BIO 14 | 31.055 | 0.000 | 0.177 | 0.000 |
| 45583 | 0 | BIO 15 | 108.099 | 0.000 | 0.177 | 0.000 |
| 45583 | 0 | RSR | 65.128 | 0.000 | 0.177 | 0.000 |
| 45583 | 0 | ASRQH | 24.701 | 0.000 | 0.177 | 0.000 |
| 45583 | 0 | LIP | 0.780 | 0.000 | 0.177 | 0.000 |
| 45583 | 0 | OP | 0.157 | 0.000 | 0.177 | 0.375 |
| 964 | 50 | BIO 2 | 0.561 | -0.001 | 0.004 | 0.000 |
| 964 | 50 | BIO 5 | 0.510 | -0.001 | 0.004 | 0.000 |
| 964 | 50 | BIO 6 | 0.246 | -0.001 | 0.004 | 0.000 |
| 964 | 50 | BIO 12 | 0.194 | -0.001 | 0.004 | 0.000 |
| 964 | 50 | BIO 14 | 0.242 | -0.001 | 0.004 | 0.000 |
| 964 | 50 | BIO 15 | 0.374 | -0.001 | 0.004 | 0.000 |
| 964 | 50 | RSR | 0.220 | -0.001 | 0.004 | 0.000 |
| 964 | 50 | ASRQH | 0.416 | -0.001 | 0.004 | 0.000 |
| 964 | 50 | LIP | 0.054 | -0.001 | 0.004 | 0.000 |
| 964 | 50 | OP | 0.061 | -0.001 | 0.004 | 0.000 |
| 509 | 100 | BIO 2 | 0.513 | -0.002 | 0.007 | 0.000 |
| 509 | 100 | BIO 5 | 0.425 | -0.002 | 0.007 | 0.000 |
| 509 | 100 | BIO 6 | 0.249 | -0.002 | 0.007 | 0.000 |
| 509 | 100 | BIO 12 | 0.201 | -0.002 | 0.007 | 0.000 |
| 509 | 100 | BIO 14 | 0.245 | -0.002 | 0.007 | 0.000 |
| 509 | 100 | BIO 15 | 0.333 | -0.002 | 0.007 | 0.000 |
| 509 | 100 | RSR | 0.265 | -0.002 | 0.007 | 0.000 |
| 509 | 100 | ASRQH | 0.356 | -0.002 | 0.007 | 0.000 |
| 509 | 100 | LIP | 0.059 | -0.002 | 0.007 | 0.000 |
| 509 | 100 | OP | 0.076 | -0.002 | 0.007 | 0.000 |
| 373 | 150 | BIO 2 | 0.473 | -0.003 | 0.009 | 0.000 |
| 373 | 150 | BIO 5 | 0.377 | -0.003 | 0.009 | 0.000 |
| 373 | 150 | BIO 6 | 0.262 | -0.003 | 0.009 | 0.000 |
| 373 | 150 | BIO 12 | 0.168 | -0.003 | 0.009 | 0.000 |
| 373 | 150 | BIO 14 | 0.223 | -0.003 | 0.009 | 0.000 |
| 373 | 150 | BIO 15 | 0.342 | -0.003 | 0.009 | 0.000 |
| 373 | 150 | RSR | 0.286 | -0.003 | 0.009 | 0.000 |
| 373 | 150 | ASRQH | 0.322 | -0.003 | 0.009 | 0.000 |
| 373 | 150 | LIP | 0.063 | -0.003 | 0.009 | 0.000 |
| 373 | 150 | OP | 0.083 | -0.003 | 0.009 | 0.000 |
| 292 | 200 | BIO 2 | 0.428 | -0.004 | 0.011 | 0.000 |
| 292 | 200 | BIO 5 | 0.313 | -0.004 | 0.011 | 0.000 |
| 292 | 200 | BIO 6 | 0.248 | -0.004 | 0.011 | 0.000 |
| 292 | 200 | BIO 12 | 0.169 | -0.004 | 0.011 | 0.000 |
| 292 | 200 | BIO 14 | 0.211 | -0.004 | 0.011 | 0.000 |
| 292 | 200 | BIO 15 | 0.290 | -0.004 | 0.011 | 0.000 |
| 292 | 200 | RSR | 0.272 | -0.004 | 0.011 | 0.000 |
| 292 | 200 | ASRQH | 0.309 | -0.004 | 0.011 | 0.000 |
| 292 | 200 | LIP | 0.055 | -0.004 | 0.010 | 0.000 |
| 292 | 200 | OP | 0.068 | -0.004 | 0.010 | 0.000 |
| 246 | 250 | BIO 2 | 0.411 | -0.005 | 0.012 | 0.000 |
| 246 | 250 | BIO 5 | 0.298 | -0.005 | 0.012 | 0.000 |
| 246 | 250 | BIO 6 | 0.247 | -0.005 | 0.012 | 0.000 |
| 246 | 250 | BIO 12 | 0.174 | -0.005 | 0.012 | 0.000 |
| 246 | 250 | BIO 14 | 0.200 | -0.005 | 0.012 | 0.000 |
| 246 | 250 | BIO 15 | 0.282 | -0.005 | 0.012 | 0.000 |
| 246 | 250 | RSR | 0.274 | -0.005 | 0.012 | 0.000 |
| 246 | 250 | ASRQH | 0.303 | -0.005 | 0.012 | 0.000 |
| 246 | 250 | LIP | 0.063 | -0.005 | 0.012 | 0.000 |
| 246 | 250 | OP | 0.057 | -0.005 | 0.012 | 0.000 |
