# Supplementary material for: Broad-scale factors shaping the ecological niche and geographic distribution of Spirodela polyrhiza
Source: PLoS One. 2023 May 4;18(5):e0276951. doi: 10.1371/journal.pone.0276951 (PMC10159170; doi:10.1371/journal.pone.0276951)
Supplement: S3 Table — (DOCX) [file pone.0276951.s029.docx]

S3 Table. Description of ecological importance of variables used for ecological niche modeling exercises with *Spirodela polyrhiza*.

| Variable (group) | Resolution | Ecological importance |
| --- | --- | --- |
| Temperature variables:   - Annual mean temperature (BIO 1) - Mean diurnal range (BIO 2) - Isothermality (BIO 3) - Temperature seasonality (BIO 4) - Max temperature of warmest month (BIO 5) - Min temperature of coldest month (BIO 6) - Temperature annual range (BIO 7) - Mean temperature of warmest quarter (BIO 10) - Mean temperature of coldest quarter (BIO 11) | 10’ and 30’ | Optimal growing temperatures range around 23°C. Temperature not only determines population growth, but at extreme levels it triggers the production of special types of fronds that help the plant to overcome cold conditions during the winter. This is why not only average temperatures, but other forms of this variable that indicate extremes and seasonality are important. |
| Precipitation variables:   - Annual precipitation (BIO 12) - Precipitation of wettest month (BIO 13) - Precipitation of driest month (BIO 14) - Precipitation seasonality (BIO 15) - Precipitation of wettest quarter (BIO 16) - Precipitation of driest quarter (BIO 17) | 10’ and 30’ | Precipitation is a variable directly related to availability of water in an area. In the absence of specifics layers indicating availability of lentic water bodies, this variable constitutes a good proxy to areas where water availability may be a limiting factor. |
| Solar radiation variables:   - Annual mean solar radiation (AMSR) - Maximum solar radiation of the month with maximum values (SRMax) - Minimum solar radiation of the month with minimum values (SRMin) - Range of solar radiation (RSR) - Average solar radiation of the quarter with highest values (ASRQH) - Average solar radiation of the quarter with lowest values (ASRQL) | 10’ and 30’ | Solar radiation is a variable that can be directly related to light intensity and availability. Light is a factor that has significant effects on this species growth rates. Understanding average values across significant periods, as well as extremes and seasonality of this variable can help to understand how suitable distinct areas are for the species. |
| Soil variables group 1:   - Cation exchange capacity (CEC) - Organic carbon (OC) - pH | 10’ | Nutrient level and availability is directly related with how populations of this species grow. The soil variables used here are proxies to characterize potential nutrient availability and level in water bodies in the areas for modeling. Phosphorus layers and OC directly indicate availability and level of these nutrients. CEC and pH inform about potential nutrient exchange dynamics. |
| Soil variables group 2:   - Total phosphorus (TP) - Labile inorganic phosphorus (LIP) - Organic phosphorus (OP) | 30’ |  |
