# Supplementary material for: Broad-scale factors shaping the ecological niche and geographic distribution of Spirodela polyrhiza
Source: PLoS One. 2023 May 4;18(5):e0276951. doi: 10.1371/journal.pone.0276951 (PMC10159170; doi:10.1371/journal.pone.0276951)
Supplement: S4 Table — AIC/AICc values are not comparable across distinct calibration areas. (DOCX) [file pone.0276951.s030.docx]

S4 Table. Selected parameter settings and variables after model calibration for analyses with variables at 10’ resolution. AIC/AICc values are not comparable across distinct calibration areas.

| Algorithm | Calibration area | Variables | Response | Regularization multiplier | pROC | Omission rates | AIC/AICc |
| --- | --- | --- | --- | --- | --- | --- | --- |
| Maxent | Buffer | BIO 5, BIO 15, ASRQH, pH | Quadratic, product | 5 | 0.00 | 0.533 | 1233.98 |
|  | Concave | BIO 5, BIO 14, ASRQH, pH | Linear, quadratic | 2.5 | 0.00 | 0.210 | 2489.18 |
|  | Ecoregions | ASRQH, pH | Quadratic, product | 0.1 | 0.00 | 0.060 | 9675.78 |
|  | Intersection | BIO 5, ASRQH, CEC, OC, pH | Quadratic | 0.1 | 0.00 | 0.049 | 17370.23 |
| GLM | Buffer | BIO 2, BIO 5, BIO 12, BIO 14, RSR, ASRQH, CEC | Linear,  Quadratic, product | - | 0.00 | 0.042 | 24582.17 |
|  | Concave | BIO 2, BIO 5, BIO 6, BIO 12, BIO 14, ASRQH, CEC | Linear, quadratic, product | - | 0.00 | 0.050 | 24402.23 |
|  | Ecoregions | BIO 2, BIO 5, BIO 6, BIO 14, BIO 15, CEC, OC | Linear, quadratic, product | - | 0.00 | 0.050 | 24210.55 |
|  | Intersection | BIO 2, BIO 5, BIO 14, BIO 15, ASRQH, CEC, OC | Linear, quadratic, product | - | 0.00 | 0.048 | 24587.30 |
