# Supplementary material for: Broad-scale factors shaping the ecological niche and geographic distribution of Spirodela polyrhiza
Source: PLoS One. 2023 May 4;18(5):e0276951. doi: 10.1371/journal.pone.0276951 (PMC10159170; doi:10.1371/journal.pone.0276951)
Supplement: S5 Table — AIC/AICc values are not comparable across distinct calibration areas. (DOCX) [file pone.0276951.s031.docx]

S5 Table. Selected parameter settings and variables after model calibration for analyses with variables at 30’ resolution. AIC/AICc values are not comparable across distinct calibration areas.

| Algorithm | Calibration area | Variables | Response | Regularization multiplier | pROC | Omission rates | AIC/AICc |
| --- | --- | --- | --- | --- | --- | --- | --- |
| Maxent | Buffer | BIO 2, BIO 5, BIO 6, BIO 12, BIO 14, RSR, OP | Linear, product | 0.1 | 0.00 | 0.045 | 14770.39 |
|  | Concave | BIO 2, BIO 6, BIO 12, BIO 14, RSR, ASRQH, OP | Linear, product | 0.1 | 0.00 | 0.048 | 14824.19 |
|  | Ecoregions | BIO 2, BIO 6, BIO 12, RSR, ASRQH, LIP, OP | Linear, product | 0.1 | 0.00 | 0.048 | 14820.13 |
|  | Intersection | BIO 5, BIO 6, BIO 12, BIO 14, RSR, ASRQH, OP | Linear, quadratic, product | 0.1 | 0.00 | 0.043 | 14627.09 |
| GLM | Buffer | BIO 6, BIO 12, BIO 14, BIO 15, RSR, ASRQH | Linear, product | - | 0.00 | 0.037 | 20290.85 |
|  | Concave | BIO 5, BIO 6, BIO 12, BIO 14, RSR, ASRQH | Linear, product | - | 0.00 | 0.047 | 20236.67 |
|  | Ecoregions | BIO 6, BIO 12, BIO 14, BIO 15, RSR, ASRQH | Linear, product | - | 0.00 | 0.045 | 20232.22 |
|  | Intersection | BIO 6, BIO 12, BIO 14, BIO 15, RSR, ASRQH | Linear, product | - | 0.00 | 0.040 | 20148.68 |
