# Supplementary material for: Broad-scale factors shaping the ecological niche and geographic distribution of Spirodela polyrhiza
Source: PLoS One. 2023 May 4;18(5):e0276951. doi: 10.1371/journal.pone.0276951 (PMC10159170; doi:10.1371/journal.pone.0276951)
Supplement: S6 Table — Results for models created with variables at 10’ resolution, using buffer calibration areas are shown. Quadratic = “^2”; Product = “:”. (DOCX) [file pone.0276951.s032.docx]

S6 Table. Effects of predictors on GLMs produced using variables and parameter settings selected after model calibration. Results for models created with variables at 10’ resolution, using buffer calibration areas are shown. Quadratic = “^2”; Product = “:”.

|  | Degrees of freedom (Df) | Deviance | Residual Df | Residual Deviance | *P* (>*Chi*) |
| --- | --- | --- | --- | --- | --- |
| NULL | - | - | 21768 | 25535 | - |
| BIO 2 | 1 | 233.312 | 21767 | 25301 | 1.13E-52 |
| CEC | 1 | 175.326 | 21761 | 24966 | 5.08E-40 |
| BIO 14 | 1 | 114.795 | 21764 | 25182 | 8.72E-27 |
| BIO 12^2 | 1 | 113.914 | 21758 | 24806 | 1.36E-26 |
| BIO 2:BIO 5 | 1 | 57.173 | 21753 | 24688 | 3.99E-14 |
| BIO 2:BIO 14 | 1 | 49.283 | 21751 | 24620 | 2.22E-12 |
| BIO 5^2 | 1 | 43.828 | 21759 | 24920 | 3.59E-11 |
| CEC^2 | 1 | 41.210 | 21754 | 24745 | 1.37E-10 |
| BIO 5:BIO 12 | 1 | 27.858 | 21747 | 24570 | 1.31E-07 |
| RSR | 1 | 22.679 | 21763 | 25159 | 1.91E-06 |
| BIO 2:BIO 12 | 1 | 18.756 | 21752 | 24669 | 1.49E-05 |
| BIO 5:ASRQH | 1 | 18.618 | 21744 | 24549 | 1.60E-05 |
| BIO 14^2 | 1 | 17.758 | 21757 | 24788 | 2.51E-05 |
| ASRQH | 1 | 17.529 | 21762 | 25141 | 2.83E-05 |
| BIO 2:ASRQH | 1 | 13.262 | 21749 | 24599 | 2.71E-04 |
| BIO 14:CEC | 1 | 9.274 | 21736 | 24514 | 2.32E-03 |
| BIO 14:ASRQH | 1 | 7.789 | 21737 | 24523 | 5.26E-03 |
| BIO 12:CEC | 1 | 7.441 | 21739 | 24533 | 6.37E-03 |
| BIO 2:RSR | 1 | 7.353 | 21750 | 24612 | 6.70E-03 |
| BIO 5:CEC | 1 | 5.096 | 21743 | 24543 | 2.40E-02 |
| RSR:ASRQH | 1 | 2.765 | 21735 | 24511 | 9.63E-02 |
| BIO 12 | 1 | 2.531 | 21765 | 25296 | 1.12E-01 |
| BIO 5 | 1 | 2.414 | 21766 | 25299 | 1.20E-01 |
| BIO 5:RSR | 1 | 2.257 | 21745 | 24567 | 1.33E-01 |
| ASRQH^2 | 1 | 2.253 | 21755 | 24786 | 1.33E-01 |
| BIO 2^2 | 1 | 2.204 | 21760 | 24964 | 1.38E-01 |
| BIO 14:RSR | 1 | 2.163 | 21738 | 24531 | 1.41E-01 |
| BIO 2:CEC | 1 | 1.551 | 21748 | 24598 | 2.13E-01 |
| BIO 12:BIO 14 | 1 | 1.431 | 21742 | 24542 | 2.32E-01 |
| BIO 12:RSR | 1 | 1.298 | 21741 | 24541 | 2.55E-01 |
| ASRQH:CEC | 1 | 0.638 | 21733 | 24510 | 4.24E-01 |
| RSR:CEC | 1 | 0.491 | 21734 | 24511 | 4.84E-01 |
| BIO 5:BIO 14 | 1 | 0.198 | 21746 | 24569 | 6.57E-01 |
| BIO 12:ASRQH | 1 | 0.039 | 21740 | 24541 | 8.44E-01 |
| RSR^2 | 1 | 0.002 | 21756 | 24788 | 9.68E-01 |
