# Supplementary material for: Broad-scale factors shaping the ecological niche and geographic distribution of Spirodela polyrhiza
Source: PLoS One. 2023 May 4;18(5):e0276951. doi: 10.1371/journal.pone.0276951 (PMC10159170; doi:10.1371/journal.pone.0276951)
Supplement: S12 Table — Results for models created with variables at 30’ resolution, using calibration areas from ecoregions are shown. Quadratic = “^2”; Product = “:”. (DOCX) [file pone.0276951.s038.docx]

S12 Table. Effects of predictors on GLMs produced using variables and parameters settings selected after model calibration. Results for models created with variables at 30’ resolution, using calibration areas from ecoregions are shown. Quadratic = “^2”; Product = “:”.

|  | Degrees of freedom (Df) | Deviance | Residual Df | Residual Deviance | *P* (>*Chi*) |
| --- | --- | --- | --- | --- | --- |
| NULL | - | - | 20048 | 21574 | - |
| BIO 14 | 1 | 337.51 | 20045 | 21135 | 2.22E-75 |
| BIO 15 | 1 | 239.55 | 20044 | 20895 | 4.94E-54 |
| ASRQH | 1 | 115.98 | 20042 | 20736 | 4.79E-27 |
| BIO 14:ASRQH | 1 | 107.65 | 20029 | 20385 | 3.20E-25 |
| BIO 12:BIO 14 | 1 | 69.78 | 20039 | 20582 | 6.64E-17 |
| BIO 6 | 1 | 60.89 | 20047 | 21513 | 6.03E-15 |
| BIO 6:BIO 14 | 1 | 50.30 | 20040 | 20652 | 1.32E-12 |
| RSR | 1 | 43.19 | 20043 | 20852 | 4.97E-11 |
| BIO 12 | 1 | 40.45 | 20046 | 21472 | 2.02E-10 |
| BIO 12:ASRQH | 1 | 38.53 | 20030 | 20493 | 5.39E-10 |
| BIO 6:BIO 12 | 1 | 33.67 | 20041 | 20702 | 6.53E-09 |
| BIO 12:BIO 14:BIO 15:RSR | 1 | 33.15 | 20002 | 20183 | 8.54E-09 |
| BIO 14:BIO 15 | 1 | 28.09 | 20036 | 20548 | 1.16E-07 |
| BIO 6:BIO 14:RSR | 1 | 27.85 | 20021 | 20304 | 1.31E-07 |
| BIO 6:BIO 14:BIO 15:ASRQH | 1 | 23.69 | 19999 | 20146 | 1.13E-06 |
| BIO 6:BIO 12:BIO 15 | 1 | 23.53 | 20025 | 20338 | 1.23E-06 |
| RSR:ASRQH | 1 | 20.42 | 20027 | 20362 | 6.20E-06 |
| BIO 14:BIO 15:RSR:ASRQH | 1 | 19.56 | 19992 | 20110 | 9.75E-06 |
| BIO 12:BIO 14:RSR | 1 | 13.05 | 20020 | 20291 | 3.04E-04 |
| BIO 12:BIO 15:RSR | 1 | 12.22 | 20018 | 20278 | 4.72E-04 |
| BIO 6:BIO 15:ASRQH | 1 | 12.20 | 20013 | 20252 | 4.78E-04 |
| BIO 12:BIO 14:BIO 15:ASRQH | 1 | 9.55 | 19998 | 20137 | 2.00E-03 |
| BIO 15:RSR:ASRQH | 1 | 7.85 | 20007 | 20227 | 5.08E-03 |
| BIO 6:ASRQH | 1 | 7.83 | 20031 | 20531 | 5.15E-03 |
| BIO 12:RSR | 1 | 7.49 | 20034 | 20540 | 6.20E-03 |
| BIO 6:BIO 12:BIO 15:ASRQH | 1 | 7.41 | 20000 | 20170 | 6.48E-03 |
| BIO 14:BIO 15:RSR | 1 | 6.47 | 20017 | 20272 | 1.10E-02 |
| BIO 12:BIO 14:BIO 15 | 1 | 6.42 | 20023 | 20332 | 1.13E-02 |
| BIO 12:BIO 15 | 1 | 6.34 | 20037 | 20576 | 1.18E-02 |
| BIO 12:BIO 14:RSR:ASRQH | 1 | 6.19 | 19995 | 20130 | 1.28E-02 |
| BIO 6:BIO 14:BIO 15:RSR | 1 | 5.43 | 20003 | 20216 | 1.98E-02 |
| BIO 6:BIO 12:BIO 14:ASRQH | 1 | 5.41 | 20001 | 20177 | 2.01E-02 |
| BIO 6:BIO 12:BIO 15:RSR | 1 | 5.04 | 20004 | 20221 | 2.47E-02 |
| BIO 12:BIO 15:ASRQH | 1 | 4.48 | 20012 | 20248 | 3.43E-02 |
| BIO 6:RSR:ASRQH | 1 | 4.21 | 20010 | 20240 | 4.02E-02 |
| BIO 14:RSR:ASRQH | 1 | 3.45 | 20008 | 20235 | 6.33E-02 |
| BIO 6:BIO 12:BIO 14:BIO 15:RSR:ASRQH | 1 | 3.39 | 19985 | 20104 | 6.57E-02 |
| BIO 6:BIO 14:ASRQH | 1 | 3.28 | 20015 | 20266 | 6.99E-02 |
| BIO 14:BIO 15:ASRQH | 1 | 3.08 | 20011 | 20244 | 7.93E-02 |
| BIO 6:BIO 12:ASRQH | 1 | 2.89 | 20016 | 20269 | 8.90E-02 |
| BIO 15:ASRQH | 1 | 2.74 | 20028 | 20382 | 9.76E-02 |
| BIO 12:RSR:ASRQH | 1 | 1.88 | 20009 | 20238 | 1.71E-01 |
| BIO 12:BIO 14:ASRQH | 1 | 1.30 | 20014 | 20264 | 2.55E-01 |
| BIO 12:BIO 14:BIO 15:RSR:ASRQH | 1 | 1.15 | 19986 | 20108 | 2.83E-01 |
| BIO 6:BIO 14:BIO 15:RSR:ASRQH | 1 | 0.91 | 19987 | 20109 | 3.41E-01 |
| BIO 6:RSR | 1 | 0.81 | 20035 | 20547 | 3.69E-01 |
| BIO 6:BIO 14:RSR:ASRQH | 1 | 0.66 | 19996 | 20136 | 4.16E-01 |
| BIO 6:BIO 12:BIO 14:RSR | 1 | 0.46 | 20005 | 20226 | 4.95E-01 |
| BIO 15:RSR | 1 | 0.39 | 20032 | 20539 | 5.35E-01 |
| BIO 6:BIO 12:BIO 15:RSR:ASRQH | 1 | 0.34 | 19988 | 20110 | 5.58E-01 |
| BIO 6:BIO 14:BIO 15 | 1 | 0.32 | 20024 | 20338 | 5.71E-01 |
| BIO 6:BIO 12:BIO 14:BIO 15 | 1 | 0.31 | 20006 | 20227 | 5.78E-01 |
| BIO 6:BIO 15:RSR | 1 | 0.22 | 20019 | 20290 | 6.39E-01 |
| BIO 6:BIO 12:RSR | 1 | 0.21 | 20022 | 20332 | 6.50E-01 |
| BIO 6:BIO 12:RSR:ASRQH | 1 | 0.12 | 19997 | 20137 | 7.31E-01 |
| BIO 6:BIO 12:BIO 14:BIO 15:RSR | 1 | 0.08 | 19991 | 20110 | 7.83E-01 |
| BIO 14:RSR | 1 | 0.02 | 20033 | 20540 | 8.83E-01 |
| BIO 6:BIO 12:BIO 14:BIO 15:ASRQH | 1 | 0.02 | 19990 | 20110 | 8.99E-01 |
| BIO 6:BIO 12:BIO 14 | 1 | 0.01 | 20026 | 20362 | 9.04E-01 |
| BIO 6:BIO 15 | 1 | 0.01 | 20038 | 20582 | 9.11E-01 |
| BIO 6:BIO 15:RSR:ASRQH | 1 | 0.01 | 19994 | 20130 | 9.31E-01 |
| BIO 12:BIO 15:RSR:ASRQH | 1 | 0.01 | 19993 | 20130 | 9.40E-01 |
| BIO 6:BIO 12:BIO 14:RSR:ASRQH | 1 | 0.00 | 19989 | 20110 | 9.71E-01 |
